# EBV infection-induced GPX4 promotes chemoresistance and tumor progression in nasopharyngeal carcinoma

**DOI:** 10.1038/s41418-022-00939-8

**Published:** 2022-02-01

**Authors:** Li Yuan, Shibing Li, Qiuyan Chen, Tianliang Xia, Donghua Luo, Liangji Li, Sailan Liu, Shanshan Guo, Liting Liu, Chaochao Du, Guodong Jia, Xiaoyun Li, Zijian Lu, Zhenchong Yang, Huanliang Liu, Haiqiang Mai, Linquan Tang

**Affiliations:** 1 Sun Yat-sen University Cancer Center, State Key Laboratory of Oncology in South China, Collaborative Innovation Center for Cancer Medicine, Guangdong Key Laboratory of Nasopharyngeal Carcinoma Diagnosis and Therapy, Guangzhou, Guangdong Province China; 2 Department of Nasopharyngeal Carcinoma, Sun Yat-sen University Cancer Center, Guangzhou, Guangdong Province China; 3 Department of Clinical Laboratory, The Sixth Affiliated Hospital, Sun Yat-sen University, Guangzhou, Guangdong Province China; 4 Guangdong Provincial Key Laboratory of Colorectal and Pelvic Floor Diseases, Guangdong Institute of Gastroenterology, The Sixth Affiliated Hospital, Sun Yat-sen University, Guangzhou, Guangdong Province China

**Keywords:** Microbiology, Oncogenes

## Abstract

Epstein–Barr virus (EBV) was the first oncogenic virus identified in humans. It is primarily associated with multiple lymphoid and epithelial cancers, including nasopharyngeal carcinoma (NPC). However, its association with ferroptosis and its role in cancer therapy resistance have not been fully elucidated. Here, we show that EBV infection reduces the sensitivity of NPC cells to ferroptosis by activating the p62-Keap1-NRF2 signaling pathway in conjunction with upregulation of SLC7A11 and GPX4 expression. Knockdown of endogenous GPX4 or blockade of GPX4 using a specific inhibitor enhanced the chemosensitivity of EBV-infected NPC cells. Functional studies revealed that GPX4 knockdown suppresses the proliferation and colony formation of NPC cells. Mechanistically, GPX4 interacts with the TAK1-TAB1/TAB3 complex, regulates TAK1 kinase activity, and further activates downstream MAPK-JNK and NFκB pathways. High GPX4 expression is correlated with poor clinical outcomes in patients with NPC and other cancer types. Taken together, our findings suggest that EBV infection has important effects on redox homeostasis, revealing a previously unappreciated role for GPX4 in tumor progression. This novel mechanism provides a potential new target for the treatment of EBV-related tumors.

## Introduction

Epstein–Barr virus (EBV) is a large double-stranded DNA virus that belongs to the gammaherpesvirinae subfamily [1]. As the first identified human oncogenic virus, EBV contributes to approximately 1.5% of all cases of cancer worldwide, including lymphoid and epithelial cancers [2–4]. The malignancy that is most closely associated with EBV infection is undifferentiated nasopharyngeal carcinoma (NPC), which occurs in the epithelial lining of the nasopharynx [5–7]. Chemoradiotherapy is the fundamental treatment strategy for NPC. However, the treatment of NPC still faces great challenges due to chemoresistance [8].

EBV is consistently detected in NPC patients from both endemic and non-endemic area. Delineating the cellular processes targeted by EBV is essential to understand its role in tumor initiation and progression and may contribute to the discovery of new therapeutic targets. Increasing evidence has emerged to reveal the functions of viral proteins (e.g., EBVN1 and LMP1) and small RNAs that may contribute to EBV-associated cancers [9, 10]. However, the role of EBV infection in the mechanism of chemoresistance has not been fully elucidated.

Ferroptosis, a nonapoptotic form of cell death, is featured by the excessive iron-dependent accumulation of lipid reactive oxygen species (ROS) [11–13]. The distinctive characteristics of cells undergoing ferroptosis include a series of morphological abnormalities of mitochondria, such as organelle shrinkage, condensed organelle membrane, and lessened cristae. Ferroptosis is precisely controlled via a regulatory network involving the inhibitory role of glutathione peroxidase 4 (GPX4) and cystine transporter SLC7A11 (system X_c_^-^, xCT). GPX4 helps to clear the toxic lipid hydroperoxides (LOOH), which prevents cellular damage from oxidative stress and maintains redox homeostasis [14], while the inhibition of GPX4 promotes lipid ROS-dependent ferroptosis. Cancer cells are known to develop iron addiction, which increases the ROS production as a result of cellular transformation and tumorigenesis [15]. In addition to their increased antioxidant capacity, cancer cells are rendered more susceptible to ferroptosis due to their altered redox environment. Therefore, cancer cells are more dependent on GPX4, especially following epithelial-to-mesenchymal transition (EMT) [16, 17]. Directly targeting GPX4 may serve as an efficient strategy to induce ferroptosis in cancer cells in vivo and provide a new approach for ROS manipulation-based cancer therapy.

To the best of our knowledge, the effect of EBV infection on the ferroptosis sensitivity of host cells has not been studied to date. Here, we show that EBV infection activates the p62-Keap1-NRF2 signaling axis, leading to upregulation of GPX4 and SLC7A11, and effectively reduces the ferroptosis sensitivity of NPC cells. Inhibition of GPX4 leads to enhanced chemosensitivity in EBV-infected NPC cells. Additionally, GPX4 knockdown significantly suppresses tumor cell proliferation in vitro and in vivo. We further demonstrated that GPX4 interacts with the TAK1-TAB1/TAB3 complex, regulates TAK1 kinase activity, and activates the downstream MAPK-JNK and NFκB pathways. Altogether, our findings uncovered a novel mechanism by which chemoresistance induced by EBV infection facilitates the evasion of ferroptosis, identifying GPX4 is a potential therapeutic target in NPC.

## Results

### EBV infection reduces ferroptosis in NPC cells

**Fig. 1:**
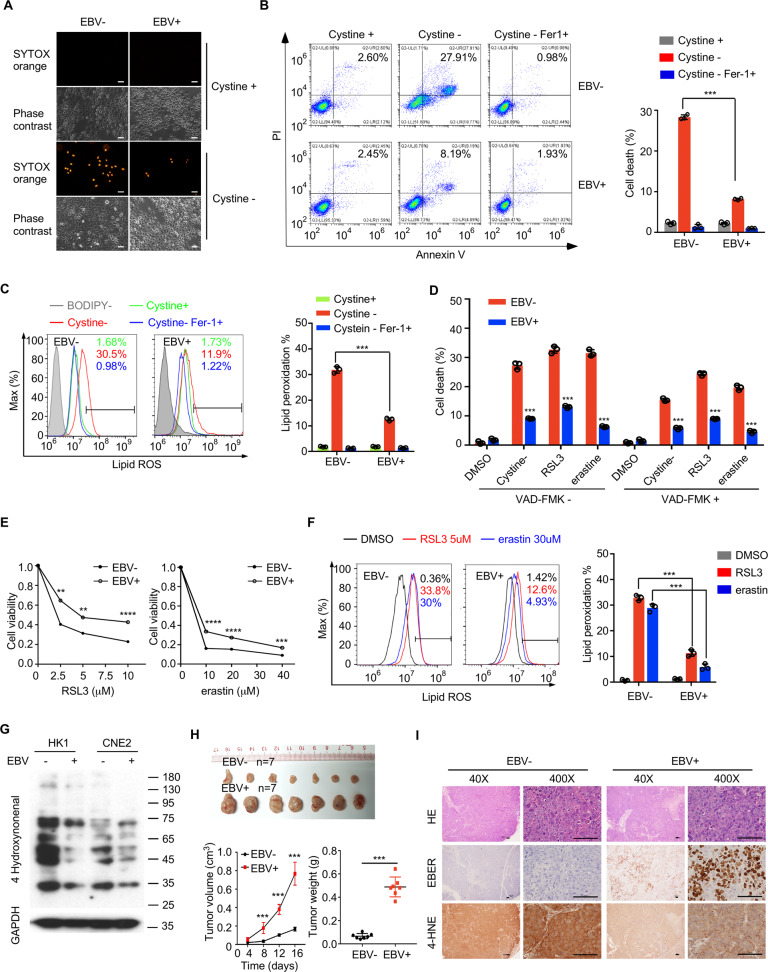
EBV infection inhibits ferroptosis in NPC cells. CNE2 EBV-negative or CNE2 EBV-positive cells were seeded into six-well plates. Cells were cultured for 24 h and then subjected to cystine starvation for 30 h. Cell death was assessed using SYTOX Orange staining (**A**) or PI-Annexin V double staining followed by flow cytometry analysis (**B**) (*n* = 3). **C** Twenty-four hours after cystine starvation and 2 μM ferrostatin-1 (Fer-1) treatment, lipid reactive oxygen species (ROS) production was determined by C11-BODIPY staining followed by flow cytometry (*n* = 3). **D** Cell death of EBV-negative or EBV-positive CNE2 cells after treatment with cystine starvation, RSL3, erastin or DMSO (control) for 30 h with or without the caspase inhibitor z-VAD-FAK (*n* = 3). **E**. Cell viability of CNE2 EBV-negative or CNE2 EBV-positive cells was determined after treatment with different concentrations of RSL3 or erastin for 30 h by CCK-8 assay (*n* = 4). **F**. Lipid ROS production in CNE2 EBV-negative or -positive cells was determined after treatment with RSL3, erastin or DMSO (control) for 24 h (*n* = 3). **G** Representative western blots of 4-hydroxynonenal in EBV-negative and EBV-positive cells. GAPDH was used as a loading control. **H** Subcutaneous tumors formed by CNE2 EBV-negative or CNE2 EBV-positive cells in nude mice were excised 17 days after inoculation. Tumor growth was assessed by volume changes over time and weight at the endpoint (*n* = 7). **I** Representative images of immunohistochemistry staining showing high levels of 4-hydroxynonena in CNE2 EBV-negative xenografts. Data are shown as the mean ± SD. **p < 0.01; ***p < 0.001; ****p < 0.0001. **B**–**F**, two-tailed unpaired t test. **H** two-tailed Mann–Whitney test. **A** and **I** scale bars: 100 µm.

To explore the effect of EBV infection on the ferroptosis sensitivity of nasopharyngeal epithelial cells, we developed EBV-infected NPC cell lines as previously described [18]. Recombinant EBV-GFP originated from the Burkitt’s lymphoma cell line Akata-EBV was introduced into two representative NPC cell lines, CNE2 and HK1. The existence of the virus was corroborated by the expression of GFP (Fig. S1A). The EBV latent infection protein EBNA1 and the lytic infection protein BZLF1 were detected in EBV-positive cells but not in EBV-negative cells (Fig. S1B). Expression of other EBV-related genes, such as LMP1, LMP2A, and BRLF1, was also confirmed (Fig. S1C, D). After cystine starvation, EBV infection conferred resistance to ferroptosis and associated lipid peroxidation, compared with EBV-negative CNE2 cell line. Furthermore, lipid ROS accumulation and cell death were diminished upon the addition of ferroptosis inhibitor ferrostatin-1 (Fer1) (Fig. 1A–C). To examine the possible involvement of apoptosis in cell death induced by cystine starvation and other ferroptosis inducer treatments, the pancaspase inhibitor z-VAD-FMK was used for 1 h before ferroptosis induction. The results showed that despite a small decrease in the percentage of dead cells in the z-VAD-FMK pre-treatment group, EBV-negative NPC CNE2 cells were still more sensitive to cell death induced by cysteine starvation, treatment with the cystine transporter inhibitor erastin or the GPX4 inhibitor RSL3 (Fig. 1D, E). Furthermore, EBV-negative CNE2 cells were more sensitive to lipid ROS accumulation induced by erastin and RSL3 (Fig. 1F). Similar results were observed in EBV-negative and -positive HK1 cell lines (Figs. S2A–F). In addition, we determined 4-hydroxynonenal (4-HNE) levels to evaluate intracellular lipid oxidation and found that EBV infection effectively reduced cellular oxidative stress (Fig. 1G). To further investigate oxidation in vivo, we assessed the subcutaneous tumor formed from EBV-negative and -positive CNE2 cells in nude mice. The xenografts were excised and assayed for oxidation by 4-HNE staining 17 days post-inoculation. EBV infection dramatically enhanced tumor growth (Fig. 1H), and the tumors stemmed from EBV-positive cells manifested lower 4-HNE levels (Fig. 1I). Taken together, our data suggest that EBV infection plays an important role in reducing ferroptotic death in NPC cells.

### Ferroptosis induced by GPX4 inhibition is not associated with EBV lytic reactivation

Oxidative stress is known to trigger or promote lytic reactivation of EBV [19]. GPX4 depletion or inhibition in EBV-infected cells may be related to the promotion of EBV lytic reactivation and associated cell death (including nonferroptosis) via enhanced oxidative stress. To test this hypothesis, EBV replication and expression levels of EBV lytic genes were assessed in GPX4-inhibited EBV-positive NPC cells. Sodium n-butyrate (SB) or 12-O-tetradecanoylphorbol-13-acetate (TPA) was used as a positive control [20]. After SB or TPA treatment for 12 h, EBV replication was examined via flow cytometry. The GFP-positive proportion of cells was increased by 30% after SB or TPA treatment (Fig. S3A). Next, cysteine starvation or treatment with RSL3 or erastin was conducted for 24 h in EBV-positive CNE2 or HK1 cells, and results revealed that only cystine starvation, but not RSL3 or erastin treatment, was correlated with the promotion of EBV lytic reactivation (Fig. S3B). We also examined expression levels of EBV latent and lytic genes, and similar results were observed (Fig. S3C). Because cystine starvation has been reported to trigger different types of cell death [21] and treatment with RSL3 or erastin represents more specific ferroptosis induction, these data indicate that cell death resulting from GPX4 inhibition in EBV-infected cells does not comprise a nonferroptotic portion caused by EBV lytic reactivation.

### EBV infection activates the p62-Keap1-NRF2 pathway and upregulates GPX4 expression in NPC cells

**Fig. 2:**
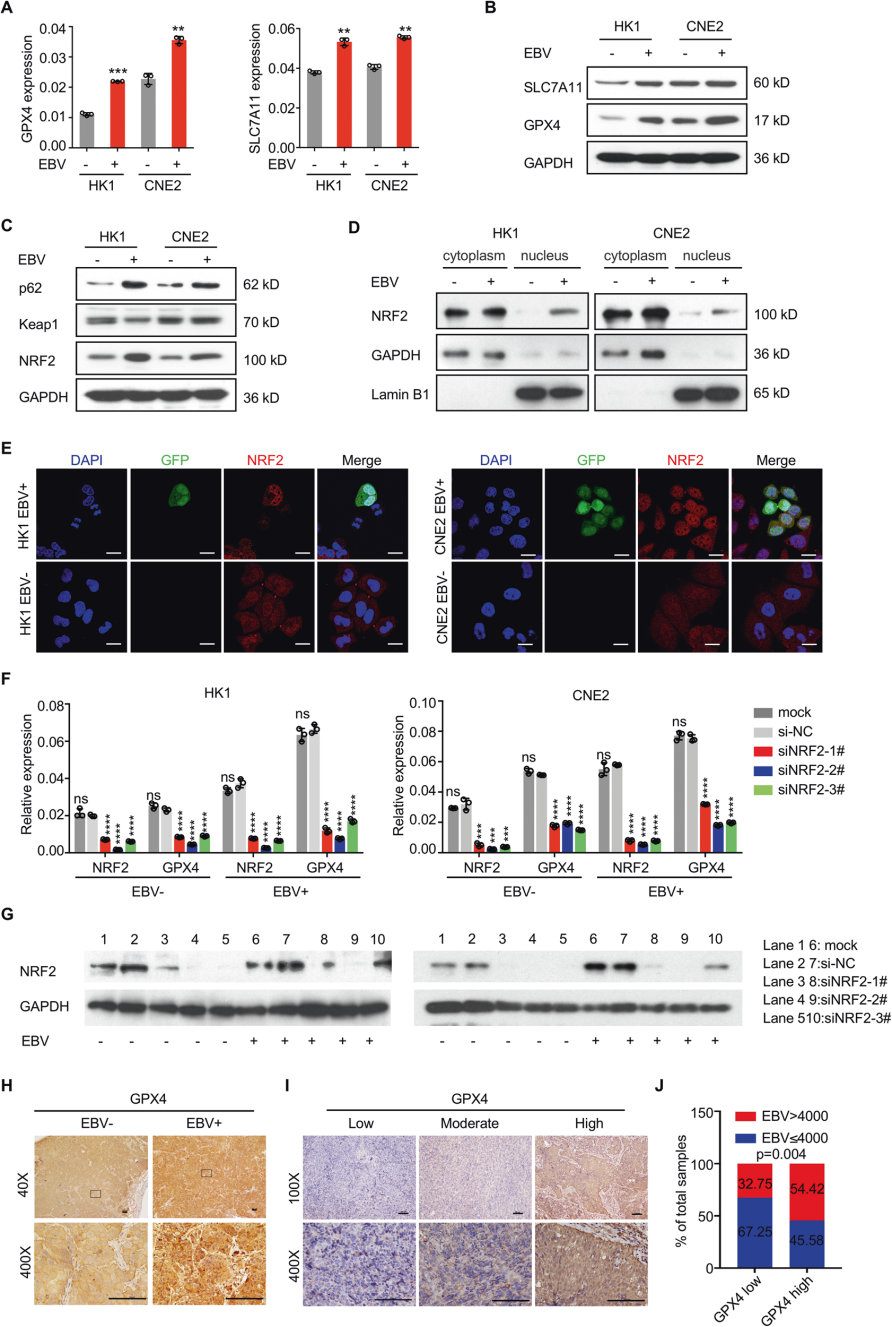
EBV infection activates the p62-Keap1-NRF2 signaling pathway and induces high GPX4 expression in NPC cells. mRNA and protein expression levels of GPX4 and SLC7A11 in EBV-negative and EBV-positive NPC cells were determined by RT–qPCR (**A**) (*n* = 3) and immunoblotting (**B**) (*n* = 3). **C** The p62-Keap1-NRF2 pathway was examined in EBV-negative and EBV-positive NPC cells by immunoblotting. **D** Cytoplasmic and nuclear proteins from EBV-negative and EBV-positive NPC cells were fractionated and detected by immunoblotting. Lamin B1 and GAPDH were used as controls for the nuclear and cytoplasmic fractions, respectively. **E** Immunofluorescence staining showing the localization of NRF2 in EBV-negative and EBV-positive NPC cells. mRNA and protein levels of NRF2 and GPX4 in EBV-negative and EBV-positive NPC cells were determined by RT–qPCR (**F**) (*n* = 3) and immunoblotting (**G**) (*n* = 3) after siRNA knockdown of endogenous NRF2. **H** GPX4 was highly expressed in CNE2 EBV + xenografts. **I** Representative images of immunohistochemistry staining showing GPX4 expression in paraffin-embedded tumor sections from NPC patients. **J** GPX4 expression in different groups according to EBV copy number in 181 NPC patients. Data are shown as the mean ± SD. **p < 0.01; ***p < 0.001; ****p < 0.0001; ns, not significant. **A**, **F** Two-tailed unpaired t test (**F**, compared to si-NC). **J** two-tailed Mann–Whitney test. **S**cale bars: 20 µm (**E**) and 100 µm (**H**, **I**).

The cystine transporter SLC7A11 and peroxidase GPX4 are key effectors of ferroptosis [22]. Both genes were upregulated in EBV-positive cells at both the mRNA (Fig. 2A) and protein levels (Fig. 2B). Nuclear factor (erythroid-derived 2)-like 2 (NRF2) is the major modulator that negatively regulates ferroptosis by increasing iron storage and inhibits ROS production [23]. Under stress-free conditions, low levels of NRF2 are sustained through proteasomal degradation mediated by Kelch-like ECH-associated protein 1 (Keap1). While under oxidative stress, the selective autophagy receptor and ubiquitin (Ub) sensor p62 binds to Keap1 and interferes with the Keap1‐NRF2 interaction when exposed to ferroptosis‐inducing agents [24], thus the NRF2 protein is stabilized and triggers downstream signal transduction to dampen the ferroptosis sensitivity of cells [25] [26, 27]. We found that EBV-positive cells exhibited remarkably higher p62 protein levels than isogenic EBV-negative cells. Consistently, EBV infection reduced Keap1 expression and resulted in elevated NRF2 levels (Fig. 2C). It further showed that NRF2 translocated more into the nucleus of EBV-positive NPC cells rather than EBV-negative cells (Fig. 2D and E). To further confirm that the p62-Keap1-NRF2 axis is responsible for the high expression of GPX4 in EBV-infected cells, we examined the effect of NRF2 on GPX4 expression in EBV-negative and EBV-positive NPC cells using siRNAs against NRF2. qRT–PCR and immunoblotting results revealed reduced expression of GPX4 in response to NRF2 knockdown (Fig. 2F and G), suggesting that upregulated NRF2 in EBV-infected cells accounts for the increased levels of GPX4. Next, we assessed GPX4 expression in NPC xenografts and found that xenografts originating from EBV-positive NPC cells expressed higher levels of GPX4 (Fig. 2H). To evaluate the correlation between EBV infection and GPX4 expression in clinical samples, we collected pre-treatment tissues from 181 NPC patients and performed GPX4 immunohistochemical staining (Fig. 2I). Patients were divided based on their EBV DNA levels (>4000 vs. ≤4000 copies/ml). Patients with high EBV DNA level presented with high GPX4 expression, consistent with the conclusion that EBV infection induces high expression of GPX4 (Fig. 2J). Taken together, these findings suggest that EBV infection activates the p62-Keap1-NRF2 pathway in NPC cells and induces the expression of GPX4.

### The EBV latent gene EBNA1 is required for induction of NRF2/GPX4 and ferroptosis inhibition

**Fig. 3:**
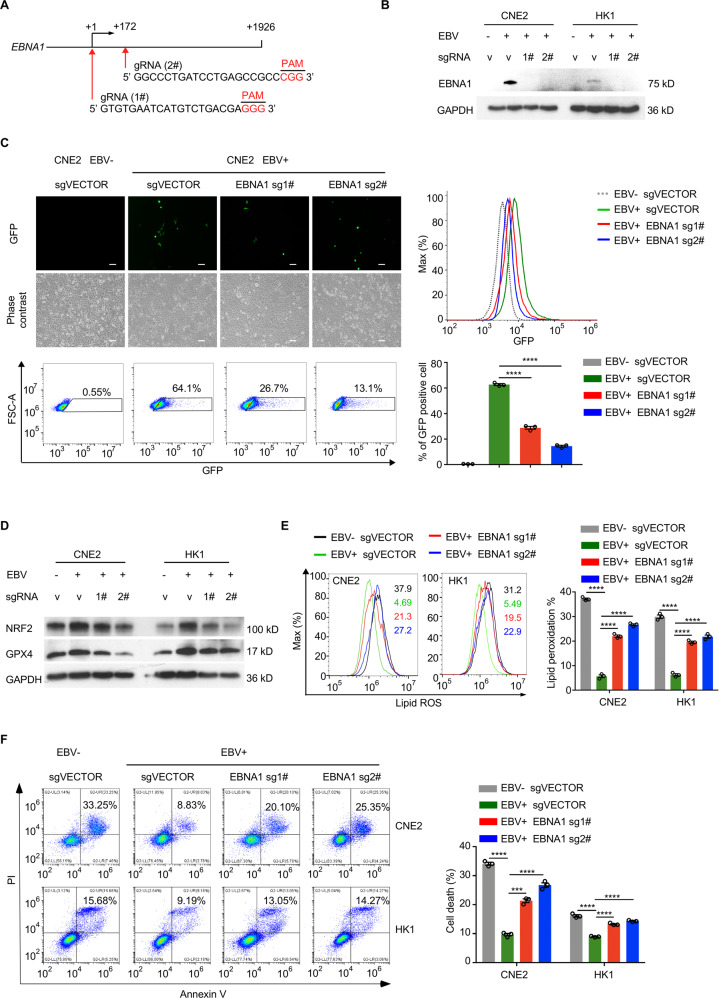
Clearance of EBV genomes enhances the sensitivity of NPC cells to ferroptosis. **A**. Two gRNA sequences targeting the EBNA1 gene of EBV. **B** EBNA1 protein expression was examined in EBV-positive NPC cells transduced with control or EBNA1-targeting gRNAs. **C** CNE2 cells carrying recombinant EBV-GFP virions were transduced with control or EBNA1-targeting gRNAs and assayed for GFP expression by fluorescence microscopy and flow cytometry (*n* = 3). **D** NRF2 and GPX4 signaling in CRISPR/Cas9-mediated EBV-negative and CRISPR/Cas9-positive NPC cells was examined by immunoblotting. **E** Twenty-four hours after 5 µM RSL3 treatment, lipid ROS production was determined by C11-BODIPY staining and flow cytometry (*n* = 3). **F** Cell death of CNE2 sgVector, CNE2-EBV sgEBNA1 or HK1 sgVector, HK1-EBV sgEBNA1 was measured and quantified after 5 µM RSL3 treatment for 24 h by flow cytometry (*n* = 3). Data are shown as the mean ± SD. ***p < 0.001; ****p < 0.0001. **C**, **E**, and **F**, two-tailed unpaired t test. **C**, scale bar, 100 µm.

Epstein–Barr nuclear antigen 1 (EBNA1) has been reported to participate in the latent and lytic EBV infection, such as interacting with specific DNA sequences in the EBV episomes which contributes to the steady genomes persistence of EBV in the process of latent infection [28]. To determine the effects of latent gene products on GPX4 gene expression and ferroptosis inhibition, EBNA1 was deleted in CNE2 and HK1 EBV-positive cell lines via CRISPR/Cas9 system. Two specific guide RNAs (gRNAs) targeting different regions of EBNA1 were used [29] (Fig. 3A) and the downregulation of EBNA1 was verified (Fig. 3B). Introducing EBNA1-targeting gRNAs into EBV-positive CNE2 cells resulted in substantially reduced GFP fluorescent signals (Fig. 3C). NRF2 and GPX4 expression was assessed and the results showed that levels of both protein were decreased after EBNA1 deletion compared to the sgVECTOR control (Fig. 3D). On the other hand, levels of lipid ROS and cell death were increased in EBNA1-deleted cells treated with RSL3 for 24 hours (Fig. 3E, F). Collectively, these results indicate that the latent gene EBNA1 is required for the induction of NRF2/GPX4 and ferroptosis inhibition.

### GPX4 is associated with chemoresistance in EBV-infected NPC cells and adverse clinical outcomes in cancer patients

**Fig. 4:**
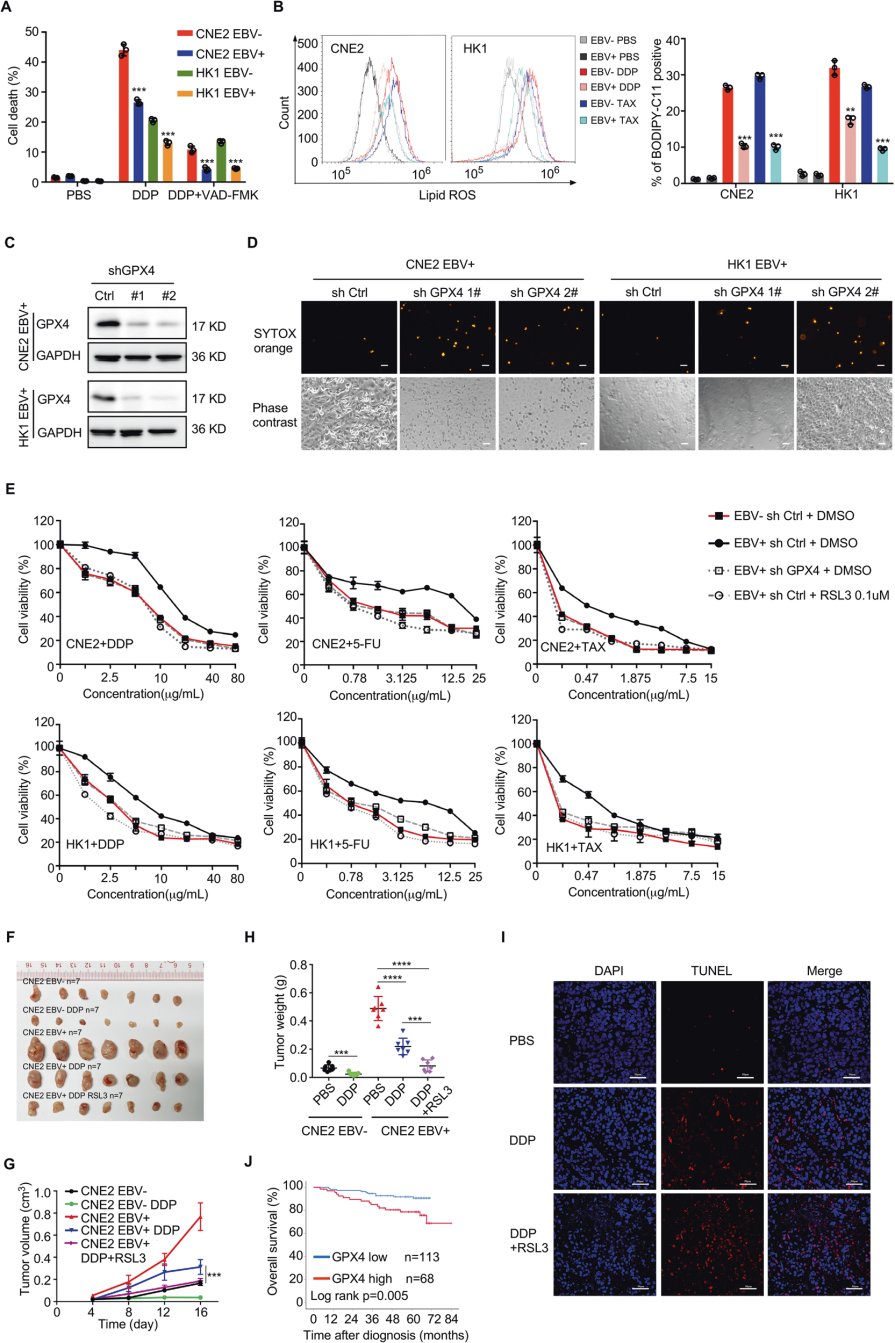
High GPX4 expression promotes chemotherapy resistance in NPC cells and is correlated with poor survival in NPC patients. **A**. Cell death of EBV-negative and EBV-positive NPC cells was determined by flow cytometry after treatment with cisplatin (DPP) with or without the caspase inhibitor VAD-FAK or PBS (control) (*n* = 3). **B** Lipid ROS production in the indicated cells was determined by flow cytometry after treatment with DDP, paclitaxel (TAX), or PBS (control) (*n* = 3). **C** Representative immunoblots of GPX4 in EBV-positive CNE2 and HK1 cells with stable knockdown of endogenous GPX4. **D** Thirty hours after cystine starvation, cell death was assessed by SYTOX Orange staining. **E** Dose–response curve for DPP, 5-fluorouracil (5-FU), and TAX treatment with or without the GPX4 inhibitor RSL3 for 48 h in the indicated cells (*n* = 4). **F**. Subcutaneous tumors formed by EBV-negative and EBV-positive CNE2 cells in nude mice were excised 17 days after inoculation. DDP (4 mg/kg) or RSL3 (10 mg/kg) was administered 4 days post inoculation. **G, H**. Growth curve and weight of xenograft tumors (*n* = 7). **I** TUNEL staining (red signal) assessing cell death in xenograft tumors. Scale bar, 50 µm. **J** Kaplan–Meier analysis of overall survival (OS) based on GPX4 expression in 181 NPC patients. Data are shown as the mean ± SD. **p < 0.01; ***p < 0.001. **A** and **B**, two-tailed unpaired t test. **H** two-tailed Mann–Whitney test. **D** scale bar: 100 µm.

Chemotherapy usually induces high ROS levels in tumor cells, eventually leading to cell death. DDP triggered not only apoptosis but also non-apoptotic death, which can be inhibited by EBV infection (Fig. 4A). To explore whether chemotherapy causes ferroptosis in NPC cells, lipid ROS levels were measured and quantified after treatment with DPP and paclitaxel (TAX). The results showed that both DDP and TAX led to lipid ROS accumulation in NPC cells, whereas EBV infection attenuated lipid ROS accumulation (Fig. 4B). To explore whether the reduced ferroptosis sensitivity of NPC cells caused by EBV infection depends on the upregulated expression of GPX4, we used two specific shRNAs to knockdown endogenous GPX4 (Fig. 4C) and found that GPX4 knockdown rendered EBV-positive cells more susceptible to ferroptosis induced by cystine starvation (Fig. 4D). We next investigated whether high GPX4 expression is involved in the chemotherapy resistance of EBV-positive NPC cells. Indeed, GPX4 knockdown or combined utilization of the low-dose GPX4 inhibitor RSL3 with DDP, 5-FU, or TAX displayed a higher inhibitory effect in EBV-positive NPC cells (Fig. 4E). To verify this conclusion in vivo, nude mice with xenografts of EBV-negative and -positive CNE2 cells were treated with DDP + DMSO or DDP + RLS3. We found that the EBV-positive CNE2 cells had a higher proliferation rate and exhibited reduced sensitivity to DDP than EBV-negative cells. Additionally, coadministration of RSL3 improved the antitumor effect of DDP (Fig. 4F–H). TUNEL staining was performed to assess cell death in xenograft tumors, and the results showed that DDP + RLS3 induced more cell deaths than DDP alone (Fig. 4I). These results suggest that high GPX4 expression contributes to chemoresistance of NPC and that its inhibition enhances the chemotherapy sensitivity of EBV-positive NPC cells. To assess the clinical relevance of our findings, we assessed the correlation between GPX4 protein levels and clinicopathological parameters in 181 NPC patients (Fig. 2I). Patients were classified into two groups based on the score for GPX4 intensity and the high score group was associated with significantly worse 5-year overall survival (90.7% vs. 78.7%, *p* = 0.005) (Fig. 4J). According to Gene Expression Profiling Interactive Analysis (GEPIA) analysis based on TCGA data, expressions of GPX4 were universally upregulated across various cancer types, and high GPX4 was associated with adverse clinical outcomes in patients with different cancers, including head and neck cancer (Fig. S4A–C). These observations demonstrate that high GPX4 expression reduces the chemosensitivity of EBV-positive NPC cells and is associated with poorer prognosis in multiple cancer patients.

### GPX4 promotes proliferation and colony formation in NPC cells

**Fig. 5:**
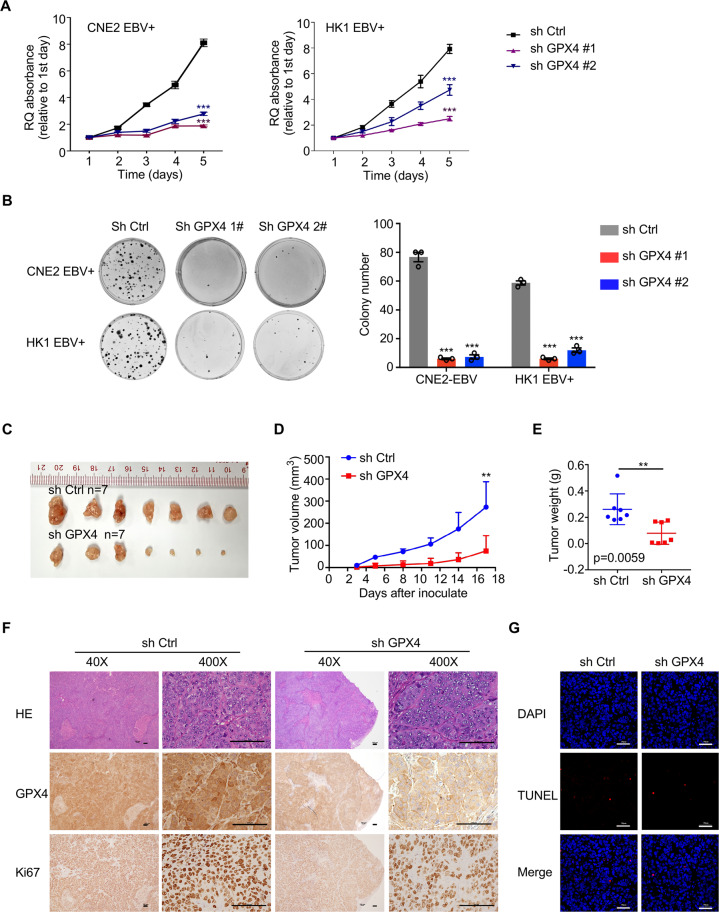
GPX4 promotes cancer cell proliferation and tumorigenicity in vitro and in vivo. **A** CCK-8 assay of EBV-positive CNE2 (left) and HK1 (right) GPX4 knockdown cells. **B** Colony formation by the indicated cells (*n* = 3). **C–E**. Subcutaneous tumors formed by EBV-positive control and shGPX4 CNE2 cells in nude mice were excised 17 days after inoculation. Tumor growth was assessed by assessing volume changes over time (**D**) and weight at the endpoint (**E**) (*n* = 7). **F** Expression of GPX4 and Ki67 in the xenografts was examined by immunohistochemistry staining. **G** Cell death in the xenograft tumors was assessed by TUNEL staining (red). Data are shown as the mean ± SD. **p < 0.01; ***p < 0.001. **A** and **B** two-tailed unpaired t test. **D** and **E** two-tailed Mann–Whitney test. **F** and **G** scale bars: 100 µm (**F**) and 50 µm (**G**).

In vitro results showed that the proliferation of EBV-positive cells with GPX4 knockdown was greatly reduced even in the absence of ferroptosis inducers. This suggests that GPX4 may act as an oncogene in addition to its peroxidase activity and metabolic regulating functions. To explore the biological function of GPX4 in NPC, we performed CCK-8 and colony formation assay and found that GPX4 knockdown significantly inhibited cell proliferation, as well as formed fewer and smaller colonies, compared to control cells (Fig. 5A and B). Cell cycle analysis and EdU incorporation assays further indicated that GPX4 knockdown inhibited the cell cycle as indicated by significantly fewer cells observed in S phase (Figs. S5A and B). We next examined the effect of GPX4 on tumorigenic potential in a xenograft model of NPC. GPX4 knockdown dramatically alleviated subcutaneous tumor burden and diminished tumor growth over time compared to the control (Fig. 5C-E). In accordance with these results, the IHC staining demonstrated a reduced intensity of the proliferation marker Ki67 in GPX4 knockdown tumor lesions (Fig. 5F). TUNEL staining revealed the same levels of cell death in xenografts originating from GPX4-knockdown NPC cells and control cells (Fig. 5G). These in vitro and in vivo results indicated the important role of GPX4 in promoting tumor progression by enhancing the capacities of proliferation and colony formation.

### GPX4 interacts with the TAK1-TAB complex

**Fig. 6:**
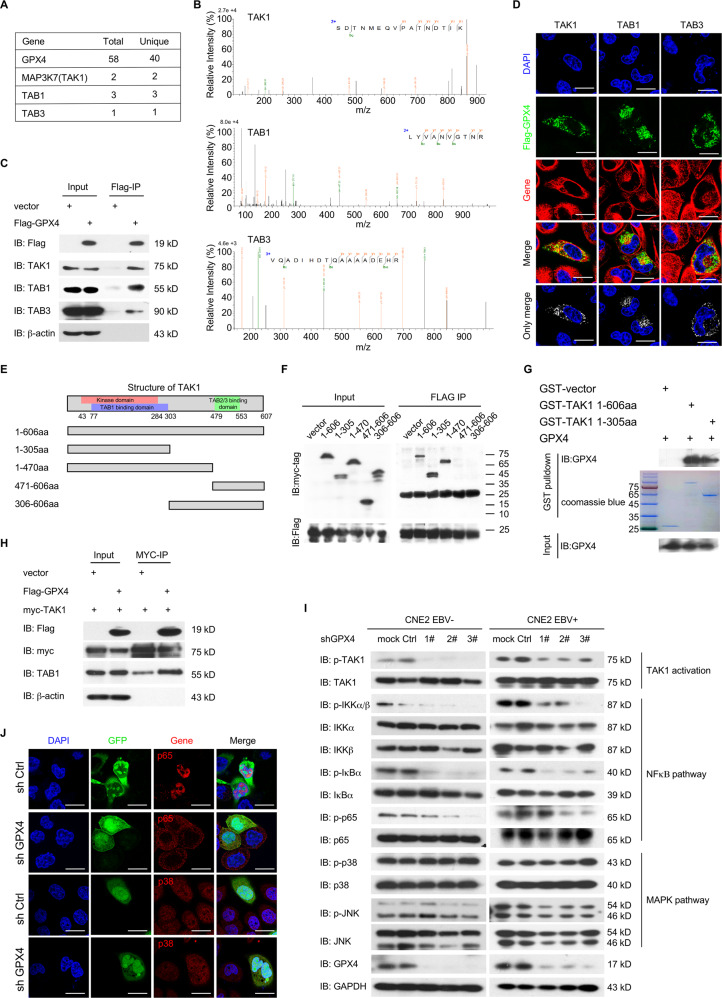
GPX4 physically interacts with the TAK1-TAB complex. **A** A partial list of interacting proteins identified by mass spectrometry using cells stably expressing GPX4. The unique and total peptide numbers for the indicated proteins are shown. **B** Representative peptides of TAK1, TAB1, and TAB3. **C** 293 T cells transfected with empty vector control or Flag-GPX4 for 48 h were subjected to the co-IP assay. **D** Representative immunofluorescence images showing the colocalization of GPX4 and the indicated genes in CNE2 cells. **E** Schematic diagram showing the structure of the TAK1 protein and the designs of different truncations for domain mapping. **F** 293 T cells transfected with Flag-GPX4 and full-length or truncated myc-TAK1 for 48 h were subjected to a co-IP assay. **G** Purified GPX4 proteins were precipitated with GST-vector, GST-TAK1 1-606aa, or GST-TAK11-305aa proteins and detected by immunoblotting using anti-GPX4 antibody. GST-fusion proteins were detected by Coomassie blue staining. **H** 293 T cells transfected with the vector control or Flag-GPX4 and myc-TAK1 expression plasmids for 48 h were subjected to co-IP assay. **I** Analysis of the TAK1-NFκB/MAPK signaling pathway in the indicated stable cell lines by immunoblotting. mock, no shRNA; Ctrl, negative control shRNA. **J** Representative immunofluorescence images of NFκB (p65) and p38 in GPX4 knockdown or control CNE2 EBV-positive cells. shCtrl, negative control shRNA. Data are representative of three biologically independent experiments. **D** and **J** Scale bars: 20 µm.

To investigate the mechanism underlying the GPX4-mediated promotion of NPC, we performed pull-down assays in combination with mass spectrometry (pull-down–MS) to explore the protein interactome of GPX4. GPX4 was found to be associated with 145 proteins, among which the TAK1-TAB complex members TAB1, TAB3, and especially MAP3K7 (also known as TAK1) attracted our attention (Fig. 6A, B).

TAK1 is a member of the mitogen-activated protein (MAP) kinase kinase kinase (MAP3K) family [30], and it functions as an intracellular hub that modulates not only MAP kinase, but also the nuclear factor-κB (NF-κB), thereby regulating multiple vital biological progresses [31]. We first confirmed the interactions between GPX4 and TAK1-TAB members (TAK1, TAB1, and TAB3) by coimmunoprecipitation (co-IP) (Fig. 6C). Additionally, immunofluorescence (IF) staining revealed that GPX4 colocalized with TAK1, TAB1, and TAB3 in the cytoplasm (Fig. 6D).

TAK1’s activation relies on the bindings with partner proteins [32], it interacts with TABs partner proteins via different domains (N-terminus for TAB1 and C-terminus for TAB2 and TAB3). To map the region of TAK1 that is necessary for GPX4 interaction, a series of TAK1 truncations encompassing known functional domains were constructed (Fig. 6E). The co-IP results showed that TAK1 1-606aa full length, 1-305aa, and 1-470aa interacted with GPX4, while 306-606aa and 471-606aa did not, indicating that the 1-305aa region of TAK1 is essential for the TAK1-GPX4 interaction (Fig. 6F). To verify whether a direct interaction occurs, we did prokaryotic expression of GPX4 and GST-TAK1 fusion protein and performed GST pull-down. Full-length GST-TAK1 (aa 1-606) and the N-terminus (aa 1-305) but not the GST-vector interacted with GPX4, suggesting that these two proteins directly interacted with each other (Fig. 6G). Considering the important role of the N-terminus in the autophosphorylation of TAK1, we speculated that GPX4 may promote the autophosphorylation and activation of TAK1. To examine the effects of GPX4 overexpression on the interactions with TAB proteins (TAK1/TAB complex formation), we transfected Flag-GPX4 and Myc-TAK1 into 293T cells and performed Myc-IP. The interaction between TAK1 and TAB1 was enhanced when GPX4 was overexpressed (Fig. 6H). To clarify the biological significance of the interaction between GPX4 and TAK1-TABs, phosphorylation of TAK1 T187 (autophosphorylation induces the activation of TAK1) and the downstream signaling effectors were assessed in EBV-negative and EBV-positive NPC cells after GPX4 knockdown. The results showed that TAK1 T187 phosphorylation and the downstream MAPK-JNK and NFκB signaling pathways were impaired in GPX4 knockdown cells. Phosphorylation levels of IKK, IκBα, and JNK were reduced in response to GPX4 knockdown, whereas MAPK-p38 phosphorylation remained unaffected (Fig. 6I). Consistent with these findings, IF staining confirmed that the nuclear translocation of p65 rather than p38 was significantly reduced after GPX4 knockdown. Taken together, these data suggest that GPX4 interacts with the TAK1-TAB complex and promotes the activation of TAK1.

### TAK1 is required for the promotion of NPC progression by GPX4

**Fig. 7:**
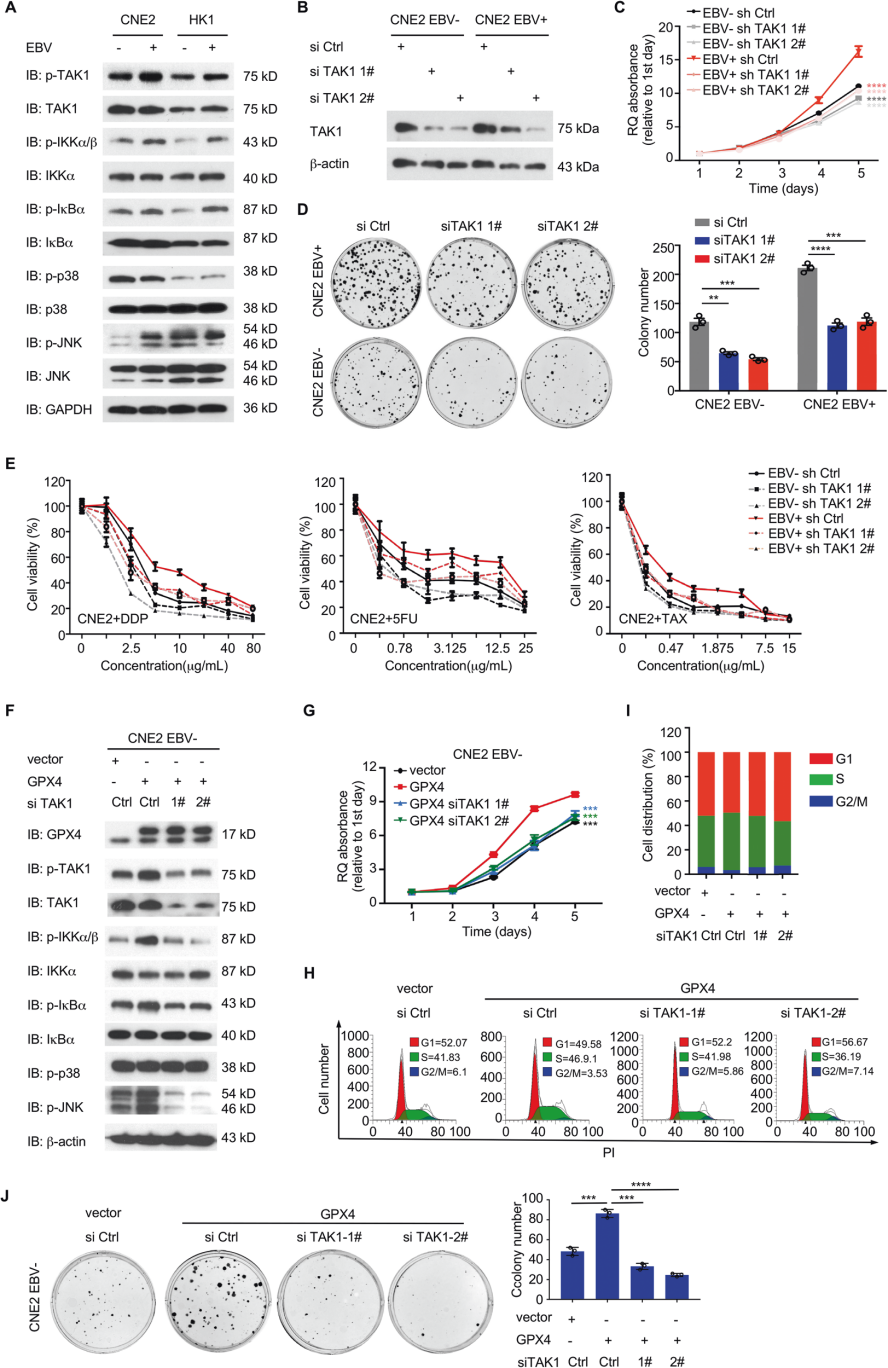
GPX4 promotes tumor progression and chemotherapy resistance in NPC by activating TAK1-JNK and IKK/NF-κB. **A**. The TAK1-NFκB/MAPK signaling pathway was examined in EBV-negative and EBV-positive NPC cells by immunoblotting**. B** Protein expression of TAK1 in EBV-negative and EBV-positive CNE2 cells transduced with siRNAs against endogenous TAK1. CCK-8 assay (**C**) and colony formation assay (**D**) in the indicated cells (*n* = 3). **E**. Dose–response curve for DPP, 5-FU, and TAX treatment in the indicated cells. **F** The TAK1-NFκB/MAPK signaling pathway was examined in the indicated stable cell lines treated with TAK1 siRNA by immunoblotting. **G** CCK-8 assay of CNE2 EBV-negative cells with stable overexpression of GPX4 treated with TAK1 siRNA (*n* = 4). **H, I**. Cell cycle analysis of the indicated cells by flow cytometry. **J** Colony formation of the indicated cells (*n* = 3). si Ctrl, negative control. Data are shown as the mean ± SD. **p < 0.01; ***p < 0.001; ****p < 0.0001. **C**, **D** and **G**, two-tailed unpaired t test.

TAK1 and downstream MAPK-JNK and NFκB signaling pathways were activated in EBV-positive NPC cells compared with EBV-negative cells, indicating the importance of EBV infection for activation of these signaling pathways (Fig. 7A). We then transfected NPC cells with TAK1-targeting siRNAs and it significantly impaired the proliferation, colony formation, and chemoresistance of NPC cells (Fig. 7B–E, S6A–D). We next investigated whether TAK1 is required for cancer-promoting effect of GPX4. TAK1-targeting siRNAs were used to transfect CNE2 EBV-negative and HK1 EBV-negative cells with stable GPX4 overexpression. As expected, the phosphorylation of TAK1 and related signaling molecules (IKK, IκBα, and JNK, but not p38) was induced with GPX4 overexpression, but decreased after TAK1 siRNA transfection. (Fig. 7F, S6E). Finally, TAK1 knockdown significantly abrogated the GPX4-mediated promotion of proliferation and colony formation in NPC cells (Fig. 7G–J, S6F–I). Taken together, these data demonstrate that TAK1 is required for the promotion of NPC cell proliferation by GPX4 in vitro.

## Discussion

As the first identified cancer-related virus, the oncogenic role of EBV is basically unequivocal for EBV-related tumors. Nonetheless, more detailed mechanisms of how EBV affects tumorigenesis and progression entail further elucidation. NPC is the most typical malignancy linked with EBV. Though curable, chemoresistance is still a critical clinical issue that greatly jeopardizes the prognosis of the disease [33, 34]. Increasing evidence indicates that EBV latent proteins and miRNAs are involved in the emergence of chemoresistance in NPC. For instance, deleting EBNA1 diminishes the abundance of EBV DNA, which in turn renders the NPC cells more sensitive to cisplatin and 5-fluorouracil [35]. LMP1 promotes chemoresistance by activating PI3K/AKT dsingling and suppressing apoptosis [36, 37]. EBV encoding miR-BART22 enhances DDP chemoresistance by targeting MAP2K4 and activating MYH9/GSK3β/β-catenin-mediated tumor stemness pathways [38]. However, the link between chemoresistance and altered redox homeostasis due to EBV infection remains largely unexplored. EBV infection has been implicated in impacting the redox homeostasis of host cells [19, 39], while chemotherapy leads to redox imbalance in tumor cells and confers higher sensitivity to ferroptosis [40]. Our results frame a model in which EBV infection contributes to ferroptosis evasion and re-establishment of redox homeostasis by upregulating GPX4 expression, facilitating the development of chemoresistance in NPC (Fig. S7). We found that EBV infection leads to upregulation of p62, which in turn activates NRF2 via the p62-Keap1-NRF2 axis in NPC cells. The downstream effectors xCT and GPX4 are upregulated and effectively reduce the accumulation of lipid ROS in tumor cells, protecting them from ferroptosis. We also revealed that GPX4 knockdown or low-dose treatment with GPX4-targeting inhibitors effectively reduces the chemoresistance of EBV-positive NPC cells, providing a potential therapeutic target for the treatment of chemoresistant tumors.

While exploring the biological functions and mechanisms of GPX4, it drew our attention that the proliferation of NPC cell lines with GPX4 knockdown decreased under conventional culture conditions without ferroptosis induction, suggesting a previously unappreciated oncogenic role of GPX4. Further in vivo and in vitro experiments confirmed this conjecture. It has been reported that peroxidases can function as pivotal regulators of various important signal molecules. In the nucleus, oligomeric peroxiredoxins (PRDXs) are directly associated with p53 or transcription factors such as NF-κB and AR, whereby cell death is regulated. Further, cytoplasmic PRDX1 modulates apoptosis via the associations with a series of ROS-dependent effectors [41, 42]. In this study, mechanistic experiments showed that GPX4 interacts with TAK1-TABs complex, among which TAK1 is particularly important as it upregulates NF-κB and MAPK signaling pathways and coordinates diverse key cellular processes. Our mass spectrometry results revealed that TAB1 and TAB3 bind with GPX4. The domain mapping of TAK1 showed that GPX4 binds to the N-terminal region of TAK1, which is the key structural region for autophosphorylation and activation of TAK1. GPX4 knockdown leads to decreased autophosphorylation of TAK1 and dampened the downstream signaling. However, knockdown of neither GPX4 nor TAK1 combined with GPX4 overexpression had a significant effect on the MAPK-p38 pathway, which suggests that the two branches of MAPK may be regulated differently in EBV-positive NPC cells.

In summary, our data reveal a novel mechanism of chemoresistance caused by GPX4 upregulation accompanied by EBV infection. The findings that EBV facilitates evasion of ferroptosis provide a proof-of-principle that may be applicable to ROS manipulation-based therapy for EBV-associated malignancies. Given the expression of GPX4 in other cancer types, its oncogenic function also highlights its potential as a therapeutic target for cancer treatment.

## Materials and methods

### Clinical specimens

To analyze GPX4 expression and GPX4-related survival, we retrospectively collected 181 paraffin-embedded NPC specimens from Sun Yat-sen University Cancer Centre between November 2010 and November 2011. All patients were diagnosed with nonmetastatic NPC, and none of the patients had received radiotherapy or chemotherapy before biopsy. The 7th edition of the AJCC Cancer Staging Manual was used to reclassify TNM stages. The median follow-up period was 62 months (range, 4–84 months). The pathologic type was WHO III in all cases. All patients received radiotherapy, and patients with stage III–IV NPC received inducing chemotherapy plus concurrent platinum-based chemotherapy or chemotherapy alone. The detailed clinicopathological characteristics are shown in Supplementary Table S1. This study was approved by the Institutional Ethical Review Board of the Sun Yat-sen University Cancer Centre (No. GZR2020-090 for human cancer specimens and NO. L102012020000N for in vivo mouse experiments). Written informed consent was obtained from all patients.

### Cell culture

Akata-EBV-GFP is an Akata Burkitt lymphoma cell line carrying the Akata bacterial artificial chromosome (BAC) with a GFP tag and was cultured in RPMI 1640 medium with 10% foetal bovine serum (FBS) (HyClone). NPC cell lines (HK1, HK1-EBV, CNE2, CNE2-EBV) were maintained in Roswell Park Memorial Institute (RPMI) 1640 medium (Life Technologies, Carlsbad, CA, USA) supplemented with 5% foetal bovine serum (FBS) in a humidified atmosphere comprising 5% CO_2_ at 37 °C. The EBV-positive Akata cell line and all NPC cell lines, which had been authenticated, were kindly provided by Dr. Mu-sheng Zeng (Sun Yat-sen University Cancer Centre). 293FT cells obtained from ATCC were grown in DMEM (Invitrogen) with 10% FBS. All cells were authenticated using short-tandem repeat profiling, tested for mycoplasma contamination, and cultured for less than 2 months.

### Antibodies and reagents

The following antibodies were used in this study: anti-4-hydroxynonenal (4-HNE), Abcam (ab46545), for immunoblotting and IHC; anti-p62, Abcam (ab109012), for immunoblotting; anti-NRF2, Abcam (ab62352), for immunoblotting and Cell Signaling Technology (#12721), for immunofluorescence staining; anti-Keap1, Abcam (ab227828), for immunoblotting; anti-SLC7A11, Abcam (ab175186), for immunoblotting; anti-GPX4, Abcam (ab125066), for immunoblotting and IHC; anti-GAPDH, Cell Signaling Technology, #5174, for immunoblotting; anti-β-actin, Abcam (ab8226), for immunoblotting; anti-TAK1, Abcam (ab109526), for immunoblotting; anti-p-TAK1(T187), Cell Signaling Technology, #4536, for immunoblotting; anti-TAB1, Abcam (ab76412), for immunoblotting and immunofluorescence staining; anti-TAB3, Abcam (ab124723), for immunoblotting and immunofluorescence staining; anti-Flag, Sigma (F1804), for immunoblotting and immunofluorescence staining; anti-IKKα, Cell Signaling Technology, #11930, for immunoblotting; anti-IKKβ, Cell Signaling Technology, #8943, for immunoblotting; anti-p-IKKα/β(Ser176/180), Cell Signaling Technology, #2697, for immunoblotting; anti-p-NFκB (Ser536), Cell Signaling Technology, #3033, for immunoblotting; anti-NFκB, Cell Signaling Technology, #8242, for immunoblotting; anti-p-IκBα (Ser32), Cell Signaling Technology, #2859, for immunoblotting; anti-IκBα (Ser32), Cell Signaling Technology, #4814, for immunoblotting; anti-p-JNK(Thr183/Tyr185), Cell Signaling Technology, #4668, for immunoblotting; anti-JNK, Cell Signaling Technology, #9252, for immunoblotting; anti-p-p38(Thr180/Tyr182), Cell Signaling Technology, #4511, for immunoblotting; and anti-p38, Cell Signaling Technology, #8690, for immunoblotting. BODIPY 581/591 C11 (Lipid Peroxidation Sensor), Invitrogen (D3861); erastin, Sigma (E7781); Ferrostatin-1, Sigma (SML0583); 1 S,3R-RSL3, Sigma (SML2234); and SYTOX Kit, Invitrogen (S34862) were also utilized.

### Virus preparation and infection

EBV with integrated EGFP (EBV-EGFP) was prepared in Akata cells as previously described [43]. The Akata-EBV-GFP cell line was used to produce EBV in accordance with the following procedure: 0.8% (v/v) goat anti-human IgG was used to treat the cells for 6 h to induce EBV into a lytic cycle, and then the cells were cultured in fresh medium for 3 days. Next, supernatants of the cells were centrifuged at 800×*g* for 30 min to remove cellular debris. Then, the supernatants were centrifuged at 50,000×g for 2 h to obtain EBV pellets. EBV pellets were resuspended in RPMI-1640 and used to infect NPC cells. EBV-infected CNE2 and HK1 cells were sorted by flow cytometry and cultured in RPMI-1640 medium using a various concentration (300–700 μg mL^−1^) of G418 (Sigma–Aldrich). EBV infection efficiency was verified by EBER detection using in situ hybridization (ISH) according to the manufacturer’s instructions of the ISH kit for EBER (Zhongshan Jinqiao Bio. Co.) or confocal laser-scanning microscopy (Olympus FV1000).

### Constructs and CRISPR/Cas9-mediated EBNA1 deletion

For EBNA1 knockout cell line construction, the plasmid lentiCRISPR-v2-EBNA1 for EBNA deletion was kindly supplied by Prof. Xiang (Xiang Tong, Sun Yat-Sen University Cancer Centre) and has been previously described [44]. For lentiviral packaging, the lentiviral vector and packaging plasmids (pMD2.G and psPAX2) were both transfected into HEK293T cells; 48 h later, the virus in the supernatant was collected and used to infect EBV-positive cells. Puromycin (2 μg mL^−1^) was added to select stable EBNA1-deleted cells. GFP-expressing cells were detected to assess EBV clearance efficiency.

### Cell death analysis

Cell death was detected using the SYTOX dead cell stain sampler kit (#S34862, Invitrogen) and the Annexin V-PI apoptosis assay kit (#556547, BD). Briefly, the indicated cells were plated in 12-well plates and treated with different concentrations of cytotoxic compounds for the indicated times. Then, SYTOX stain was added to the cell supernatants, and the cells were incubated at room temperature for 10 min, followed by observation and imaging using a fluorescence microscope. The Annexin V-PI apoptosis assay was quantified using flow cytometry.

### Lipid ROS assay

Lipid ROS production was detected by flow cytometry using C11-BODIPY dye (#D-3861, Life Technologies, Grand Island, NY, USA) according to the manufacturer’s instructions. Briefly, cells were seeded into 12-well plates and cultured in a 37 °C incubator with 5% CO_2_. After the cells were treated with different cytotoxic reagents for the indicated times, C11-BODIPY was added to the cell supernatants, and the cells were cultured for more than 30 min before ROS detection. ROS can oxidize the polyunsaturated butadienyl portion of C11-BODIPY, and then the fluorescence emission peak of the dye shifts from ~590 nm to ~510 nm.

### Oligonucleotides, plasmids, and stable cell lines

The pEZ-Lv105-puro-vector and GPX4 plasmids were purchased from GeneCopoeia, Inc., USA. The sequence of the human GPX4 gene was synthesized and cloned into the lentiviral plasmid pEZ-Lv105-puro-vector. The primers used for amplification were as follows:

GPX4-Forward 5′-ATGAGCCTCGGCCGCCTTTG-3′;

GPX4-Reverse 5′-CCCACAAGGTAGCCAGGGGT-3′.

GPX4 shRNA sequences were as follows:

shGPX4 1#: GCTACAACGTCAAATTCGA

shGPX4 2#: GAGGCAAGACCGAAGTAAA

To generate stably transfected cell lines, 293FT cells were cotransfected with the lentivirus packaging vector and shRNA knockdown plasmids. The lentiviral particles were subsequently harvested and used to infect NPC cells 48 h later. Stable clones were then selected using 1 mg/mL puromycin (Sigma–Aldrich), and real-time RT–PCR or western blot assays were used to validate the infection efficiency.

### siRNA transfection and qRT–PCR

Effective siRNA oligonucleotides targeting TAK1 and NRF2 were obtained from RiboBio with the following sequences:

siTAK1-#1: GGAGTGGCTTATCTTCACA;

siTAK1-#2: GGCTTATCTTACACTGGAT;

siNRF2-1#: GAGAAAGAATTGCCTGTAA;

siNRF2-2#: GCTACGTGATGAAGATGGA;

siNRF2-3#: GCCCTCACCTGCTACTTTA.

The negative control (siCtrl) was nonhomologous to any human genome sequence and purchased from RiboBio Co., Ltd. Predetermined cells were grown in 6-well plates for 12 h and then transfected with 20 nM siRNA mixed with 5 μL of Lipofectamine RNAiMAX (Invitrogen) according to the manufacturer’s instructions. Thirty-six to 48 h after transfection, the cells were harvested for further analysis.

Total RNA was extracted using TRIzol (Life Technology), reverse transcribed into cDNA using the GoScriptTM Reverse Transcription System (Promega) and analyzed by real-time qPCR on a BIO-RAD Real-time PCR machine using iTaq™ Universal SYBR® Green Super mix (Bio–Rad). The results were normalized to β-actin, and relative values were calculated using the 2^[-(CT gene -CT reference)]^ method. The gene-specific primers used for qPCR are listed in Supplementary Table 3.

### Western blot assay

Total protein was obtained using RIPA buffer (Beyotime Biotechnology) containing EDTA-free Protease Inhibitor Cocktail (Roche). Protein extracts were separated on 10–12% acrylamide SDS–PAGE and transferred to polyvinylidene fluoride membranes (Merck Millipore). The membranes were blocked in 5% non-fat milk and incubated with primary antibodies overnight at 4 °C. The membranes were then incubated with horseradish peroxidase-conjugated secondary antibodies (anti-mouse or anti-rabbit; 1:3,000; Thermo) at room temperature for 45 min. Finally, the target protein bands were detected using an enhanced chemiluminescence system (Thermo Fisher Scientific).

### Coimmunoprecipitation (co-IP) and mass spectrometry

For the immunoprecipitation (IP) assay, HK1 cells transfected with the empty vector or Flag-GPX4 expression plasmid were lysed in IP lysis buffer. ANTI-FLAG® M2 Affinity Gel (Sigma, A2220-10 ML) was incubated with the lysates overnight at 4 °C and then washed and collected according to the manufacturer’s protocol. Mass spectrometry was performed by Huijun Biotechnology. For the co-IP assay, western blotting was performed to determine protein levels. Cells were rinsed in PBS and then lysed in IP lysis buffer. The protein extracts were subsequently incubated with the ANTI-FLAG® M2 Affinity Gel. The precipitated proteins were separated and detected by western blotting using the indicated antibodies. Finally, the blots were visualized using a chemiluminescence system.

### GST pull-down assay

For the GST pull-down assay, recombinant human GPX4 protein expressed in Escherichia coli was purchased from Abcam company (ab82660), and GST-TAK1 (full length 1-606 aa or N-terminal 1-305 aa) was expressed in Rossita. GST-TAK1 or GST-vector and GPX4 were incubated in lysis buffer overnight at 4 °C. GST-agarose was added to lysis buffer, incubated for 4 h at 4 °C, washed four times with lysis buffer and analyzed by immunoblotting.

### Immunofluorescence staining

For immunofluorescence staining, cells were grown on coverslips (Thermo Fisher Scientific). After 24 h, the cells were fixed in 4% paraformaldehyde, permeabilized with 0.1% Triton X-100 in PBS, and incubated with primary anti-NRF2 (1:200; Cell Signaling Technology (#12721)), anti-Flag (1:200; Sigma, F1804), anti-MAP3K7 (1:1000; Abcam, ab109502), anti-TAB1 (1: 400; Abcam, ab76412), and anti-TAB3 (1: 50; Abcam, ab124723) antibodies overnight at 4 °C. The coverslips were then incubated with Alexa Fluor 488- or 594-conjugated goat IgG secondary antibodies (1:1,000; Life Technologies; A-11008 or A-11001) and counterstained with 4′,6-diamidino-2-phenylindole (DAPI). Images were captured using a confocal laser-scanning microscope (Olympus FV1000).

### IHC staining

Expression levels of GPX4 in patient tissues were detected by IHC in paraffin-embedded sections. Briefly, the sections were deparaffinized in xylene, rehydrated in a graded alcohol series, incubated in 3% hydrogen peroxide to block endogenous peroxidase activity, heated in antigen retrieval solution, subjected to FBS to block nonspecific binding, and incubated with GPX4 antibody (Abcam, ab125066, 1:100) at 4 °C overnight. Two experienced pathologists validated the scores of all sections.

### Measurement of cell death, cell viability and lipid peroxidation

Cell death was analyzed using propidium iodide (Invitrogen, Waltham, MA, USA) or SYTOX Green (Invitrogen) staining followed by microscopy or flow cytometry. Viability was measured using the CCK-8 assay. To analyze lipid peroxidation, cells were stained with 5 μM BODIPY-C11 (Invitrogen) for 30 min at 37 °C followed by flow cytometric analysis. Lipid ROS-positive cells were defined as cells with fluorescence greater than 99% of the cells in the unstained sample.

### CCK-8 assay

Cell growth was measured using a CCK-8 kit (Sigma, St Louis, MO, USA) according to the instructions. Briefly, approximately 1500 cells were plated into 96-well plates and cultured in the indicated medium. Cell proliferation measured at 450 nm (A_450_) was examined every day for five days according to the manufacturer’s protocol. All experiments were performed three times.

### Cell cycle analysis and EdU incorporation analysis

The EdU incorporation assay was performed as previously described [45]. Briefly, 5 × 10^4^ cells were seeded onto coverslips in 24-well plates. After reaching 80% confluence, EdU (20 μM) was added to the supernatants of cells and cultured for 1.5 h, followed by the click reaction according to the manufacturer’s instructions. Then, the cells were assessed under a confocal microscope to calculate the positive rate of EdU incorporation. The cell cycle was evaluated using propidium iodide (PI) staining and quantified using a Gallios flow cytometer.

### Colony formation assay

Treated cells (400 cells/well) were plated in triplicate into 6-well plates and cultured for 10 days. The colonies were fixed in methanol for 15 min and stained with crystal violet for another 15 min. Finally, the colony number was quantified. All experiments were performed three times.

### Statistical analysis

All data are presented as the means ± SD from at least three independent experiments. Two-tailed unpaired Student’s *t* test was used for statistical analysis involving two group comparisons (ns, not significant; *p < 0.05, **p < 0.01, ***p < 0.001, ****p < 0.0001). Data with a non-Gaussian distribution were compared using a two-tailed Mann–Whitney test, which was performed for the growth curves of the indicated xenografts. Differences with *p* < 0.05 were considered statistically significant. Statistical analyses were performed using GraphPad Prism (GraphPad Software, San Diego, CA). The Kaplan–Meier method and log-rank test were used to construct survival curves and compare the differences, respectively. Independent prognostic factors were determined by multivariate analysis using a Cox proportional hazards regression model. A chi-squared (χ2) test was performed to determine the correlation between EBV copy number and GPX4 expression. Statistical analyses were performed using SPSS 22.0 software (IBM).

## Supplementary information

change of authorship

English certification

Supplementary Figure Legends

Supplemental Figure 1

Supplemental Figure 2

Supplemental Figure 3

Supplemental Figure 4

Supplemental Figure 5

Supplemental Figure 6

Supplemental Figure 7

Supplementary Table 1

Supplementary Table 2

Supplementary Table 3

aj-checklist

## Data Availability

Gene-expression profiling interactive analysis in different cancer types and corresponding normal tissues were deposited at GEPIA based on TCGA and GTEx data (http://www.ncbi.nlm.nih.gov/geo/). The authenticity of this article has been validated by uploading the key raw data onto the Research Data Deposit public platform (http://www.researchdata.org.cn), with the approval RDD number as RDDB2021319938. Scripts and additional data related to this work will be available upon request to the corresponding author.
