# Supplementary material for: EBV infection-induced GPX4 promotes chemoresistance and tumor progression in nasopharyngeal carcinoma
Source: Cell Death Differ. 2022 Feb 1;29(8):1513–27. doi: 10.1038/s41418-022-00939-8 (PMC9346003; doi:10.1038/s41418-022-00939-8)
Supplement: Supplementary file 1 — change of authorship [file 41418_2022_939_MOESM1_ESM.pdf]

SPRINGER NATURE

Change of authorship request form - Journals (pre-acceptance)

Important information. Please read.

- This form should be used by authors to request any change in authorship (adding/deleting authors) including changes in corresponding authors. This form should not be used for name changes. Please fully complete all sections. Use black ink and block capitals and provide each author's full name with the given name first followed by the family name.

- By signing this declaration, all authors guarantee that the order of the authors are in accordance with their scientific contribution, if applicable as different conventions apply per discipline, and that only authors have been added who made a meaningful contribution to the work.

- Please note, in author collaborations where there is formal agreement for representing the collaboration, it is sufficient for the representative or legal guarantor (usually the corresponding author) to complete and sign the Authorship Change Form on behalf of all authors, **next to the added/removed author(s)**. **(Complete Section 3, followed by Section 6.)**
  In author collaborations where there is no formal agreement for representing the collaboration and **there are more than 10 authors**, one may sign for all, provided the signer appends correspondence that attests that each of the authors have agreed to the change **and the added/removed authors sign the form**. **(Complete Section 3, followed by Section 6.)**

- Please note, we cannot investigate or mediate any authorship disputes. If you are unable to obtain agreement from all authors (including those who you wish to be removed) you must refer the matter to your institution(s) for investigation. Please inform us if you need to do this.

- If you are not able to return a fully completed form within **30 days** of the date that it was sent to the author requesting the change, we may have to withdraw your manuscript. We cannot publish manuscripts where authorship has not been agreed by all authors (including those who have been removed).

- Incomplete forms will be rejected.

- Please return/upload this form, fully completed, to the Journals Editorial Office. The Journal and/or Publisher will consider the information you have provided to decide whether to approve the proposed change in authorship. We may decide to contact your institution for more information or undertake a further investigation, if appropriate, before making a final decision.

SPRINGER NATURE Springer Nature is one of the world's leading global research, educational and professional publishers, created in May 2015 through the combination of Nature Publishing Group, Palgrave Macmillan, Macmillan Education and Springer Science+Business Media.

SPRINGER NATURE

Change of authorship request form - Journals (pre-acceptance)

Section 1: Please provide the current title of manuscript

Manuscript ID no.: CDD-21-1060RRR

Title: EBV infection induced GPX4 promotes chemoresistance and tumor progression in nasopharyngeal carcinoma

Section 2: Please provide the previous authorship, in the order shown on the manuscript before the changes were introduced. Please indicate the corresponding author by adding (CA) behind the name.

	First name(s)	Family name	ORCID or SCOPUS id, if available
1st author	Li	Yuan	0000-0003-3935-7237
2nd author	Shibing	Li	
3rd author	Qiuyan	Chen	
4th author	Tianliang	Xia	
5th author	Donghua	Luo	
6th author	Sailan	Liu	
7th author	Shanshan	Guo	
8th author	Liting	Liu	
9th author	Chaochao	Du	
10th author	Guodong	Jia	

Please use an additional sheet if there are more than 10 authors.

SPRINGER NATURE Springer Nature is one of the world's leading global research, educational and professional publishers, created in May 2015 through the combination of Nature Publishing Group, Palgrave Macmillan, Macmillan Education and Springer Science+Business Media.

SPRINGER NATURE

Change of authorship request form - Journals (pre-acceptance)

Section 1: Please provide the current title of manuscript

Manuscript ID no.: CDD-21-1060RRR

Title: EBV infection induced GPX4 promotes chemoresistance and tumor progression in nasopharyngeal carcinoma

Section 2: Please provide the previous authorship, in the order shown on the manuscript before the changes were introduced. Please indicate the corresponding author by adding (CA) behind the name.

	First name(s)	Family name	ORCID or SCOPUS id, if available
11th author	Xiaoyun	Li	
12th author	Zhenchong	Yang	
13th author	Haiqiang	Mai CA	0000-0002-9035-6814
14th author	Linquan	Tang CA	0000-0002-9613-7305
15th author			
16th author			
17th author			
18th author			
19th author			
20th author			

Please use an additional sheet if there are more than 10 authors.

SPRINGER NATURE Springer Nature is one of the world's leading global research, educational and professional publishers, created in May 2015 through the combination of Nature Publishing Group, Palgrave Macmillan, Macmillan Education and Springer Science+Business Media.

Change of authorship request form - Journals (pre-acceptance)

Section 3: Please provide a justification for change. Please use this section to explain your reasons for changing the authorship of your manuscript, e.g. what necessitated the change in authorship? Please refer to the (journal) policy pages for more information about authorship. Please explain why omitted authors were not originally included and/or why authors were removed on the submitted manuscript.

Compared with the original submission, we proposed a new authorship. We added Dr Liangji,Li; Mr Zijian,Lu and Prof. Huanliang, Liu as the co-author of this work for their generous help and valuable advice of this study during revise period. Liangji Li and Huanliang Liu helped to revise the manuscript. Zijian, Lu participated in some experiments.

Section 4: Proposed new authorship. Please provide your new authorship list in the order you would like it to appear on the manuscript. Please indicate the corresponding author by adding (CA) behind the name. If the Corresponding Author has changed, please indicate the reason under section 3.

	First name(s)	Family name (this name will appear in full on the final publication and will be searchable in various abstract and indexing databases)	Affiliated institute	E-mail address
1st author	Li	Yuan	Sun Yat-sen University Cancer Center	yuanli@sysucc.org.cn
2nd author	Shibing	Li	Sun Yat-sen University Cancer Center	lishb26@mail.sysu.edu.cn
3rd author	Qiuyan	Chen	Sun Yat-sen University Cancer Center	chenqy@sysucc.org.cn
4th author	Tianliang	Xia	Sun Yat-sen University Cancer Center	xiatl@sysucc.org.cn
5th author	Donghua	Luo	Sun Yat-sen University Cancer Center	luodh@sysucc.org.cn
6th author	Liangji	Li	Sun Yat-sen University Cancer Center	lilj2@sysucc.org.cn
7th author	Sailan	Liu	Sun Yat-sen University Cancer Center	liusl@sysucc.org.cn
8th author	Shanshan	Guo	Sun Yat-sen University Cancer Center	guoshsh@sysucc.org.cn
9th author	Liting	Liu	Sun Yat-sen University Cancer Center	liult@sysucc.org.cn
10th author	Chaochao	Du	Sun Yat-sen University Cancer Center	duchch@sysucc.org.cn

Please use an additional sheet if there are more than 10 authors.

SPRINGER NATURE Springer Nature is one of the world's leading global research, educational and professional publishers, created in May 2015 through the combination of Nature Publishing Group, Palgrave Macmillan, Macmillan Education and Springer Science+Business Media.

SPRINGER NATURE

Change of authorshiprequest form- Journals(pre-acceptance)

Section 4: Proposed new authorship. Please provide your new authorship list in the order you would like it to appear on the manuscript. Please indicate the corresponding author by adding (CA) behind the name. If the Corresponding Author has changed, please indicate the reason under section 3

	First name(s)	Family name (this name will appear in full on the final publication and will be searchable in various abstract and indexing databases)	Affiliated institute	E-mail address
11th author	Guodong	Jia	Sun Yat-sen University Cancer Center	jiagd@sysucc.org.cn
12th author	Xiaoyun	Li	Sun Yat-sen University Cancer Center	lixy@sysucc.org.cn
13th author	Zijian	Lu	Sun Yat-sen University Cancer Center	luzj@sysucc.org.cn
14th author	Zhenchong	Yang	Sun Yat-sen University Cancer Center	yangzc@sysucc.org.cn
15th author	Huanliang	Liu	The Sixth Affiliated Hospital, Sun Yat-sen University	liuhuanl@mail.sysu.edu.cn
16th author	Haiqiang	Mai CA	Sun Yat-sen University Cancer Center	maihq@sysucc.org.cn
17th author	Linquan	Tang CA	Sun Yat-sen University Cancer Center	tanglq@sysucc.org.cn
18th author				
19th author				
20th author				

Please use an additional sheet if there are more than 10 authors.

SPRINGER NATURE Springer Nature is one of the world's leading global research, educational and professional publishers, created in May 2015 through the combination of Nature Publishing Group, Palgrave Macmillan, Macmillan Education and Springer Science+Business Media.

Change of authorship request form - Journals (pre-acceptance)

Section 5: Author contribution, Acknowledgement and Disclosures. Please use this section to provide a new disclosure statement and, if appropriate, acknowledge any contributors who have been removed as authors and ensure you state what contribution any new authors made (if applicable per the journal or book (series) policy). Please ensure these are updated in your manuscript - after approval of the change(s) - as our production department will not transfer the information in this form to your manuscript.

New acknowledgements:

The EBV-positive Akata cell line and EBV-negative and EBV-positive NPC cell lines (HK1 and CNE2) were a kind gift from Prof. Musheng Zeng (Sun Yat-sen University Cancer Centre, Guangzhou, P. R. China). The two gRNAs targeting EBNA1 were a kind gift from Dr. Tong Xiang (Sun Yat-sen University Cancer Centre, Guangzhou, P. R. China).

New Disclosures (financial and non-financial interests, funding):

This study was funded by grants from the National Key R&D Program of China (2017YFC0908500, 2017YFC1309003), the National Natural Science Foundation of China (81425018, 81672868, 81802775, 82073003, 82002852, 82003267), China Postdoctoral Science Foundation (2019M663252), the Sci-Tech Project Foundation of Guangzhou City (201707020039), the Sun Yat-sen University Clinical Research 5010 Program (2019023), the Special Support Plan of Guangdong Province (No. 2014TX01R145), the Natural Science Foundation of Guangdong Province (2017A030312003, 2018A0303131004), the Natural Science Foundation of Guangdong Province for Distinguished Young Scholars (2018B030306001), the Sci-Tech Project Foundation of Guangdong Province (2014A020212103), the Health & Medical Collaborative Innovation Project of Guangzhou City (201400000001, 201803040003), Pearl River S&T Nova Program of Guangzhou (201806010135), the Planned Science and Technology Project of Guangdong Province (2019B020230002), the National Science & Technology Pillar Program during the Twelfth Five-year Plan Period (2014BAI09B10), Natural Science Foundation of Guangdong Province (2017A030312003), and the Fundamental Research Funds for the Central Universities.

New Author Contributions statement (if applicable per the journal policy):

Linquan Tang and Haiqiang Mai conceived and designed the experiments, provided supervision and wrote the manuscript. Li Yuan, Shibing Li and Qiuyan Chen designed and performed most of the experiments, analysed the data and created the figures. Tianliang Xia, Donghua Luo, Sailan Liu, Shanshan Guo, Liting Liu, Chaochao Du, Guodong Jia, Xiaoyun Li and Zhenchong Yang participated in some experiments. Liangji Li and Huanliang Liu helped to revise the manuscript. All authors read and approved the final manuscript.

State 'Not applicable' if there are no new authors.

SPRINGER NATURE Springer Nature is one of the world's leading global research, educational and professional publishers, created in May 2015 through the combination of Nature Publishing Group, Palgrave Macmillan, Macmillan Education and Springer Science+Business Media.

SPRINGER NATURE Change ofauthorshiprequest form-Journals (pre-acceptance)

Section6: Declaration of agreement. All authors, unchanged, new and removed must sign this declaration.
(NB: Please print the form, (docu)-sign and return/upload a scanned copy. Please note that signatures that have been inserted as an image file are acceptable as long as it is handwritten. Typed names in the signature box are unacceptable.) * Please delete as appropriate. Delete all of the bold if you were on the original authorship list and are remaining as an author.

	First name	Family name		Signature	Date
1st author	Li	Yuan	I agree to the proposed new authorship shown in section 4	Li Yuan	Jan. 11. 2022
2nd author	Shibing	Li	I agree to the proposed new authorship shown in section 4	Shibing Li	Jan. 11. 2022
3rd author	Qiuyan	Chen	I agree to the proposed new authorship shown in section 4	Qiuyan Chen	Jan. 11 2022
4th authors	Tianliang	Xia	I agree to the proposed new authorship shown in section 4	Tianliang Xia	1/11/2022
5th author	Donghua	Luo	I agree to the proposed new authorship shown in section 4	Donghua Luo	1/11/2022
6th author	Liangji	Li	I agree to the proposed new authorship shown in section 4 /and the addition of my name to the authorship list	[illegible]	1/11/2022
7th author	Sailan	Liu	I agree to the proposed new authorship shown in section 4	Sailan Liu	Jan. 11. 2022

SPRINGER NATURE Springer Nature is one of the world's leading global research, educational and professional publishers, created in May 2015 through the combination of Nature Publishing Group, Palgrave Macmillan, Macmillan Education and Springer Science+Business Media.

SPRINGER NATURE

Change ofauthorshiprequest form-Journals(pre-acceptance)

	First name	Family name		Signature	Date
8th author	Shanshan	Guo	I agree to the proposed new authorship shown in section 4	Shan Shan Guo	Jan. 11. 2022
9th author	Liting	Liu	I agree to the proposed new authorship shown in section 4	Liting Liu	Jan. 11. 2022
10th author	Chaochao	Du	I agree to the proposed new authorship shown in section 4	Chaochao Du	Jan. 11. 2022
11th author	Guodong	Jia	I agree to the proposed new authorship shown in section 4	Guodong Jia	Jan. 11. 2022
12th author	Xiaoyun	Li	I agree to the proposed new authorship shown in section 4	Xiaoyun Li	Jan. 11. 2022
13th author	Zijian	Lu	I agree to the proposed new authorship shown in section 4 /and the addition of my name to the authorship list	Zijian Lu	Jan. 11. 2022
14th author	Zhenchong	Yang	I agree to the proposed new authorship shown in section 4	Zhenchong Yang	Jan. 11 22

SPRINGER NATURE Springer Nature is one of the world's leading global research, educational and professional publishers, created in May 2015 through the combination of Nature Publishing Group, Palgrave Macmillan, Macmillan Education and Springer Science+Business Media.

SPRINGER NATURE **Change ofauthorshiprequest form-Journals**(pre-acceptance)

	First name	Family name		Signature	Date
15th author	Huanliang	Liu	I agree to the proposed new authorship shown in section 4 /and the addition of my name to the authorship list	Huanliang Liu	Jan.10,2022
16th author	Haiqiang	Mai	I agree to the proposed new authorship shown in section 4	Haiqiang Mai	Jan.11,2022
17th author	Linquan	Tang	I agree to the proposed new authorship shown in section 4	Linquan Tang	Jan.11.2022

Please use an additional sheet if there are more than 10 authors.

In case of author collaborations with formal agreement:

	Name of consortium/consortia	First name	Family name		Signature	Date
Representative/legal guarantor				I agree to the proposed new authorship shown in section 4 /and the addition/removal* of my name to the authorship list/and the proposed change in corresponding author		

Both added/removed authors should complete the information in the first table under Section 6.

---- End of form ----

SPRINGER NATURE Springer Nature is one of the world's leading global research, educational and professional publishers, created in May 2015 through the combination of Nature Publishing Group, Palgrave Macmillan, Macmillan Education and Springer Science+Business Media.
