# Supplementary material for: EBV infection-induced GPX4 promotes chemoresistance and tumor progression in nasopharyngeal carcinoma
Source: Cell Death Differ. 2022 Feb 1;29(8):1513–27. doi: 10.1038/s41418-022-00939-8 (PMC9346003; doi:10.1038/s41418-022-00939-8)
Supplement: Supplementary file 2 — English certification [file 41418_2022_939_MOESM2_ESM.pdf]

SPRINGER NATURE
Author Services

Editing Certificate

This document certifies that the manuscript

EBV infection induced GPX4 promotes chemoresistance and tumor progression of nasopharyngeal carcinoma

prepared by the authors

Li Yuan1,2 †, Shibing Li1,3,4 †, Qiuyan Chen1,2 †, Tianliang Xia1, Donghua Luo2, Liangji Li1,2, Sailan Liu1,2, Shanshan Guo1,2, Liting Liu1,2, Chaochao Du1,2, Guodong Jia1,2, Xiaoyun Li1,2, Zijian Lu1,2, Zhenchong Yang1,2, Huanliang Liu3,4, Haiqiang Mai1,2* and Linquan Tang1,2*

was edited for proper English language, grammar, punctuation, spelling, and overall style by one or more of the highly qualified native English speaking editors at SNAS.

This certificate was issued on **December 21, 2021** and may be verified on the SNAS website using the verification code **D98B-BD44-4112-C1AF-9D7A**.

Neither the research content nor the authors' intentions were altered in any way during the editing process. Documents receiving this certification should be English-ready for publication; however, the author has the ability to accept or reject our suggestions and changes. To verify the final SNAS edited version, please visit our verification page at secure.authorservices.springernature.com/certificate/verify. If you have any questions or concerns about this edited document, please contact SNAS at support@as.springernature.com.

SNAS provides a range of editing, translation, and manuscript services for researchers and publishers around the world. For more information about our company, services, and partner discounts, please visit authorservices.springernature.com.
