# Supplementary material for: EBV infection-induced GPX4 promotes chemoresistance and tumor progression in nasopharyngeal carcinoma
Source: Cell Death Differ. 2022 Feb 1;29(8):1513–27. doi: 10.1038/s41418-022-00939-8 (PMC9346003; doi:10.1038/s41418-022-00939-8)
Supplement: Supplementary file 3 — Supplementary Figure Legends [file 41418_2022_939_MOESM3_ESM.docx]

**Supplementary figure legends**

**Supplementary Figure 1. EBV infection inhibits ferroptosis in NPC cells**

**A.** Representative images of EBV-negative and EBV-positive NPC cell lines. EBV infection is shown as green fluorescence. Scale bar, 100 µm. **B.** Representative western blots of EBV latent infection protein EBNA1 and lytic infection protein BZLF1 in EBV-negative and EBV-positive NPC cell lines. **C-D.** qRT–PCR of EBV latent or lytic infection genes in EBV-negative and EBV-positive NPC cell lines.

**Supplementary Figure 2. EBV infection inhibits ferroptosis in NPC cells**

**A.** EBV-negative or EBV-positive HK1 cells were seeded into six-well plates and cultured for 24 h. Thirty hours after cystine starvation, cell death was assessed by SYTOX Orange staining. **B.** Flow cytometry detecting cell death after cystine starvation (n = 3). **C.** Twenty-four hours after cystine starvation and treatment with 2 μM ferrostatin-1 (Fer-1), lipid ROS production was determined by C11-BODIPY staining followed by flow cytometry (n = 3). **D.** Cell death of EBV-negative or EBV-positive HK1 cells after treatment with cystine starvation, RSL3, erastin or DMSO (control) with or without the caspase inhibitor VAD-FAK (n = 3). **E.** Cell viability of EBV-negative or EBV-positive HK1 cells was determined after treatment with different concentrations of RSL3 or erastin by CCK-8 assay. **F.** Lipid ROS production in EBV-negative or EBV-positive HK1 cells was determined after treatment with RSL3, erastin or DMSO (control) (n = 3). Data are presented as the mean ± SD. ***p<0.001. **B**, **C**, **D**, **F** two-tailed unpaired t test. **A**, Scale bar: 100 µm.

**Supplementary Figure 3. Cystine starvation but not RSL3 or erastin treatment is related to the promotion of EBV lytic reactivation**

**A.** EBV-negative and EBV-positive CNE2 cells were treated with sodium butyrate or 12-O-tetradecanoylphorbol-13-acetate (TPA) for 12 h, and GFP expression was assessed by flow cytometry (n = 3). **B.** EBV-positive CNE2 and HK1 cells were treated with cystine starvation, RSL3 or erastin for 24 h, and GFP expression was assessed by flow cytometry. **C.** qRT–PCR of EBV latent or lytic infection genes in EBV-positive NPC cell lines treated with cystine starvation, RSL3 or erastin for 24 h. Data are shown as the mean ± SD. ****p<0.0001. **A**, two-tailed unpaired t test.

**Supplementary Figure 4. GPX4 is correlated with poor prognosis in multiple cancer types**

**A.** GPX4 expression levels in different cancers in the TCGA database. Student’s t test, -, no difference; *p<0.05; **p<0.01; ***p<0.001. BLCA, Bladder Urothelial Carcinoma; BRCA, Breast invasive carcinoma; CHOL, Cholangiocarcinoma; COAD, Colon adenocarcinoma; ESCA, Oesophageal carcinoma; GBM, Glioblastoma multiforme; HNSC, Head and neck squamous cell carcinoma; KICH, Chromophobe renal cell carcinoma; KIRC, Renal clear cell carcinoma; KIRP, Renal papillary cell carcinoma; LAML, Acute myeloid leukaemia; LGG, Brain lower grade glioma; LIHC, Hepatocellular carcinoma; LUAD, Lung adenocarcinoma; LUSC, Lung squamous cell carcinoma; PAAD, Pancreatic adenocarcinoma; PRAD, Prostate adenocarcinoma; READ, Rectal adenocarcinoma; STAD, Stomach adenocarcinoma; TGCT, Testicular germ cell tumors; THCA, Thyroid carcinoma and UCEC, Uterine corpus endometrial carcinoma. **B.** Kaplan–Meier analysis of overall survival according to GPX4 expression levels in head and neck squamous carcinoma. **C.** Kaplan–Meier analysis of overall survival according to GPX4 expression levels in different cancer types.

**Supplementary Figure 5. GPX4 knockdown impairs proliferation in EBV-positive cells**

**A.** The cell cycle status of control or GPX4 knockdown EBV-positive CNE2 and HK1 cells was determined by flow cytometry. **B.** Representative images of EdU incorporation in the indicated cells (n = 3). Data are shown as the mean ± SD. ***p<0.001. **B**, two-tailed unpaired t test. Scale bar: 20 µm.

**Supplementary Figure 6. GPX4 promotes tumor progression and chemotherapy resistance in NPC by activating TAK1-JNK and IKK/NFκB A.** Protein levels of TAK1 in EBV-negative and EBV-positive HK1 cells transduced with siRNAs against endogenous TAK1. **B-C.** CCK8 assay (**B**) and colony formation assay (**C**) in the indicated cells (n = 3). **D.** Dose–response curve for DPP, 5-FU and TAX treatment in the indicated cells (n = 3). **E.** The TAK1-NFκB/MAPK signaling pathway was examined in the indicated stable cell lines treated with TAK1 siRNA by immunoblotting. **F.** CCK8 assay of CNE2 cells with stable overexpression of GPX4 treated with siRNA (n = 3). **G-H.** Cell cycle analysis of the indicated cells by flow cytometry. **I.** Colony formation by the indicated cells (n = 3). si Ctrl, negative control siRNA. Data are shown as the mean ± SD. ***p<0.001, ****p<0.0001. **C**, **F**, and **I**, two-tailed unpaired t test.

**Supplementary Figure 7. Working model illustrating the mechanism by which EBV-induced GPX4 promotes** **chemoresistance and tumor progression in NPC.**

**Supplementary tables**

Supplementary Table 1: Patient characteristics.

Supplementary Table 2: Univariate and multivariate analysis using a Cox proportional hazards model to predict OS in NPC patients.

Supplementary Table 3: qRT–PCR primers.
