# Supplementary figures and images for: EBV infection-induced GPX4 promotes chemoresistance and tumor progression in nasopharyngeal carcinoma
Source: Cell Death Differ. 2022 Feb 1;29(8):1513–27. doi: 10.1038/s41418-022-00939-8 (PMC9346003; doi:10.1038/s41418-022-00939-8)

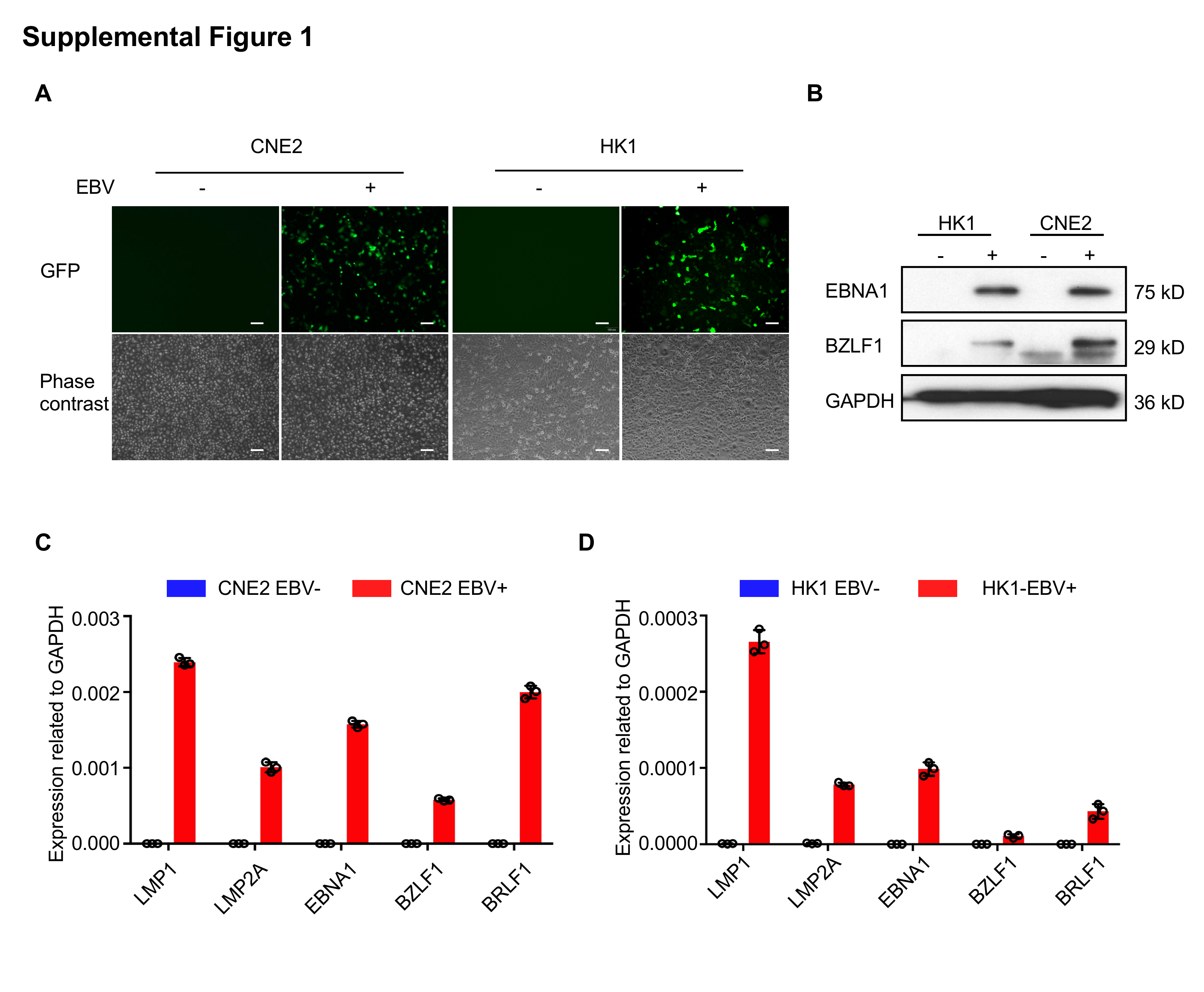

Supplement: Supplementary file 4 — Supplemental Figure 1 [file 41418_2022_939_MOESM4_ESM.png]

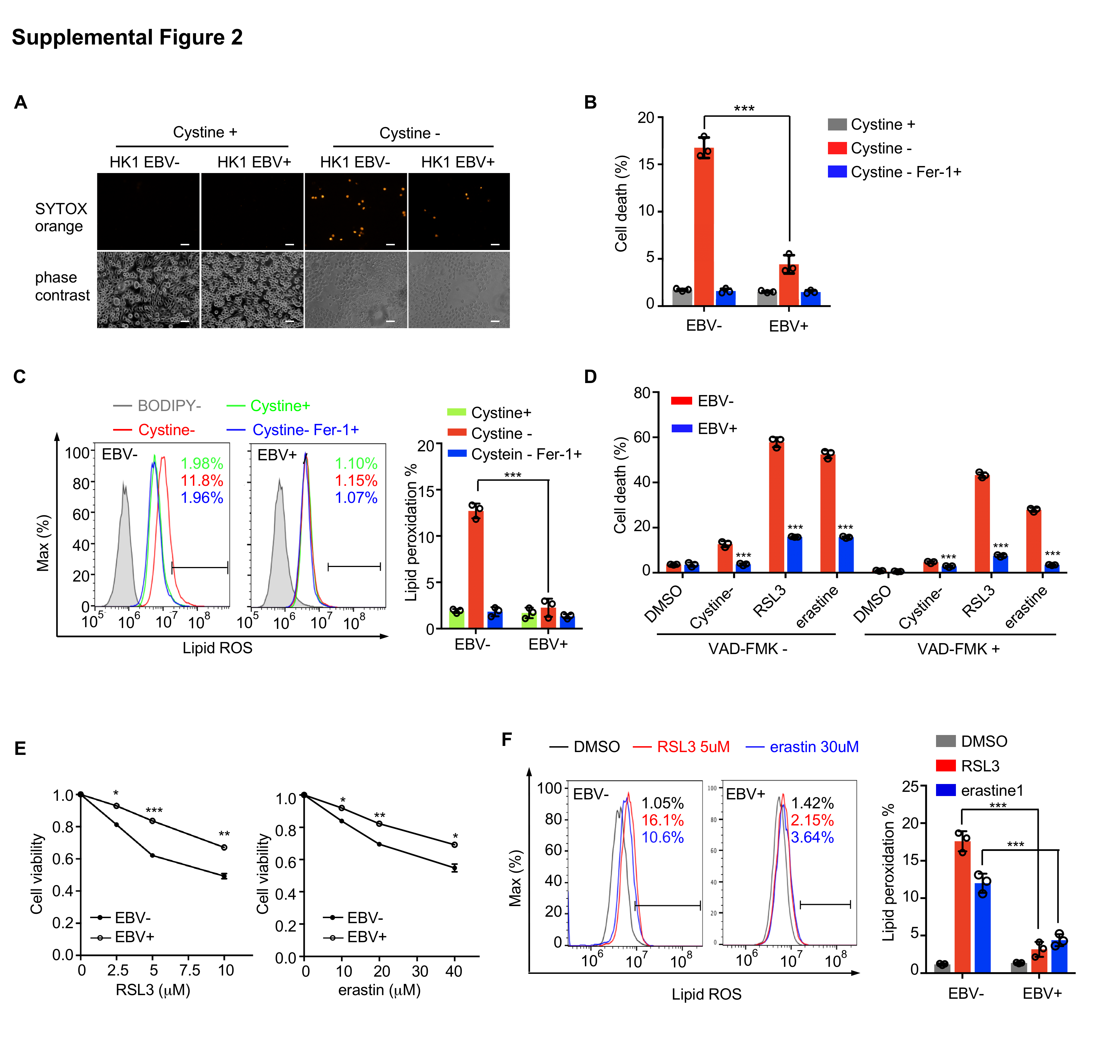

Supplement: Supplementary file 5 — Supplemental Figure 2 [file 41418_2022_939_MOESM5_ESM.png]

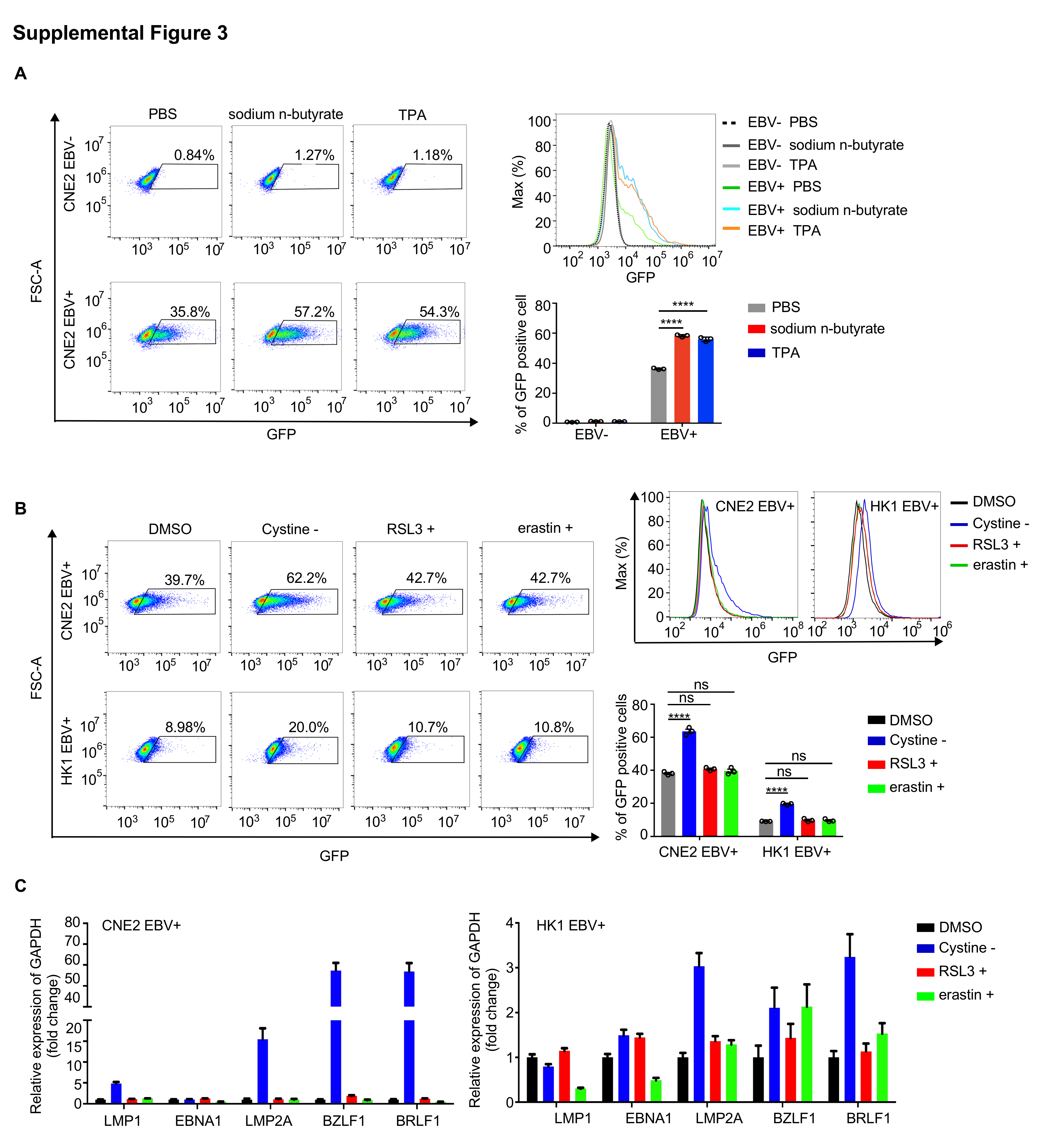

Supplement: Supplementary file 6 — Supplemental Figure 3 [file 41418_2022_939_MOESM6_ESM.png]

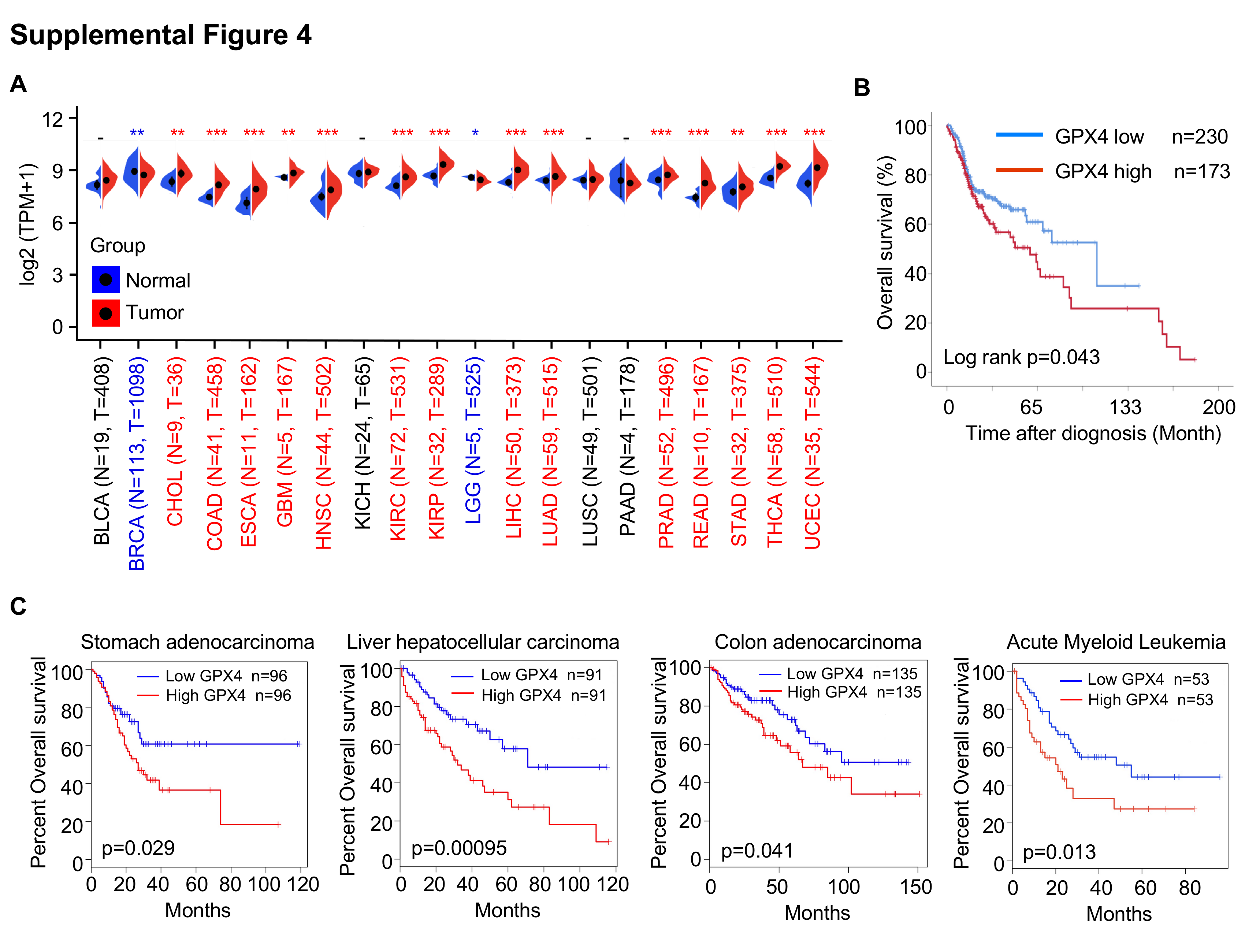

Supplement: Supplementary file 7 — Supplemental Figure 4 [file 41418_2022_939_MOESM7_ESM.png]

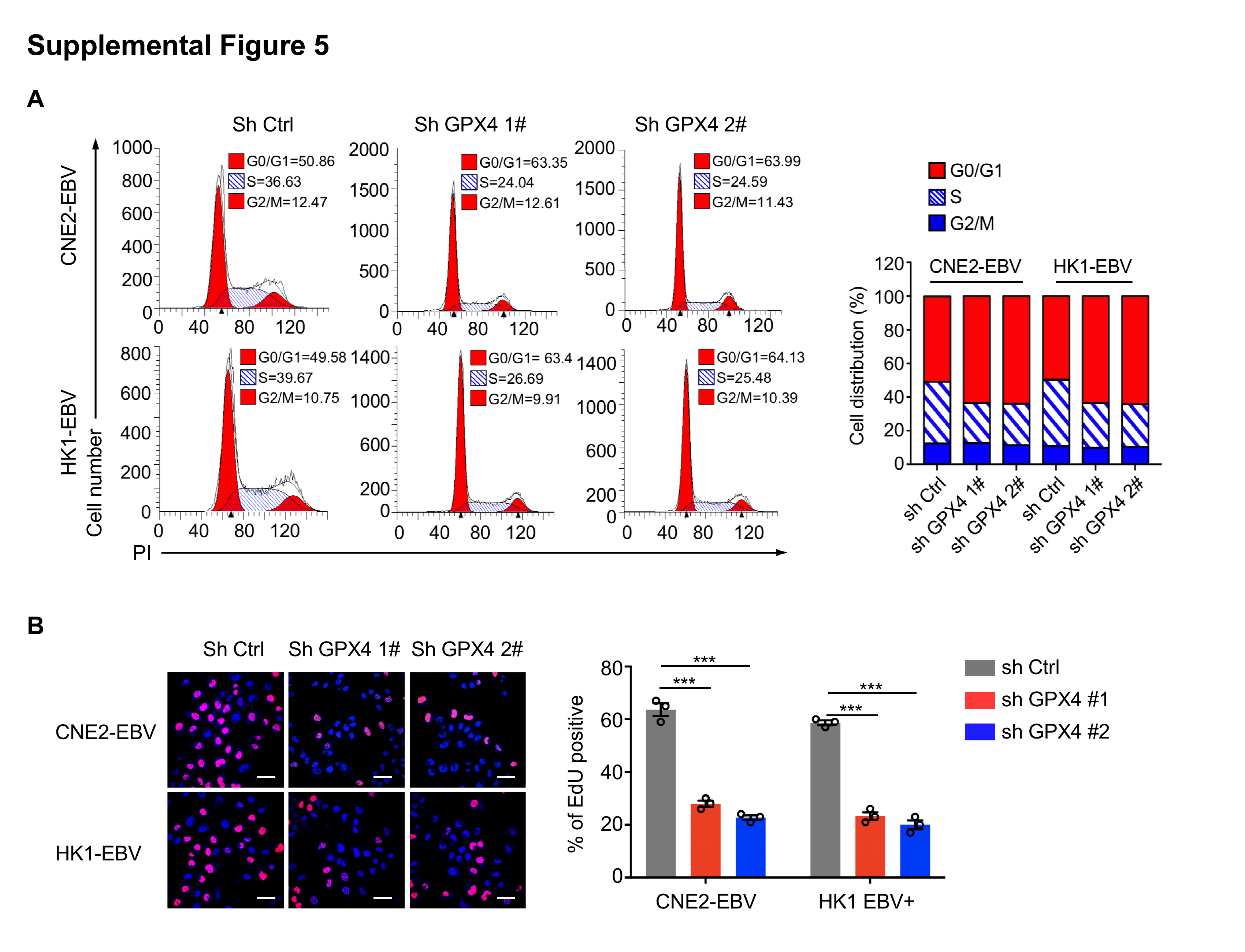

Supplement: Supplementary file 8 — Supplemental Figure 5 [file 41418_2022_939_MOESM8_ESM.png]

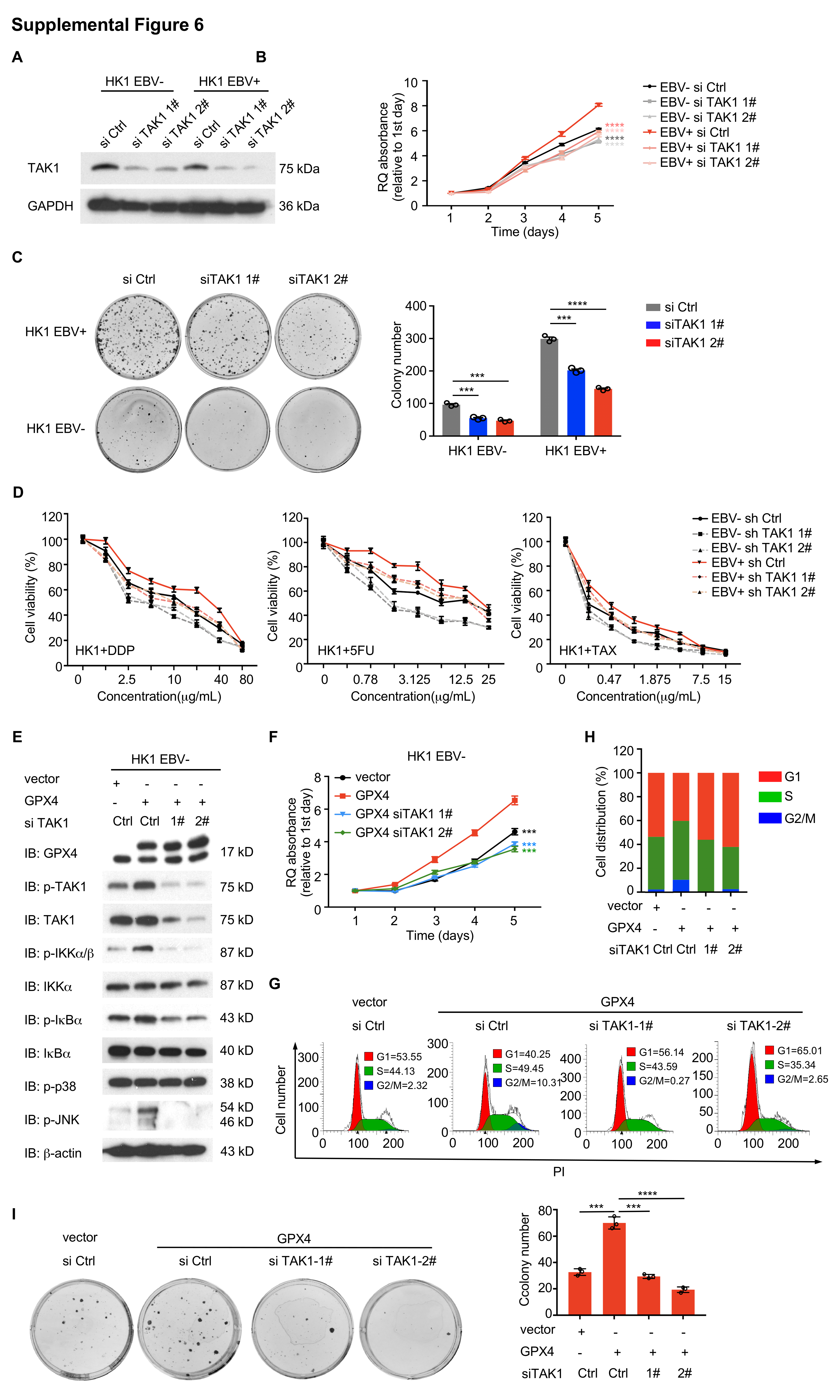

Supplement: Supplementary file 9 — Supplemental Figure 6 [file 41418_2022_939_MOESM9_ESM.png]

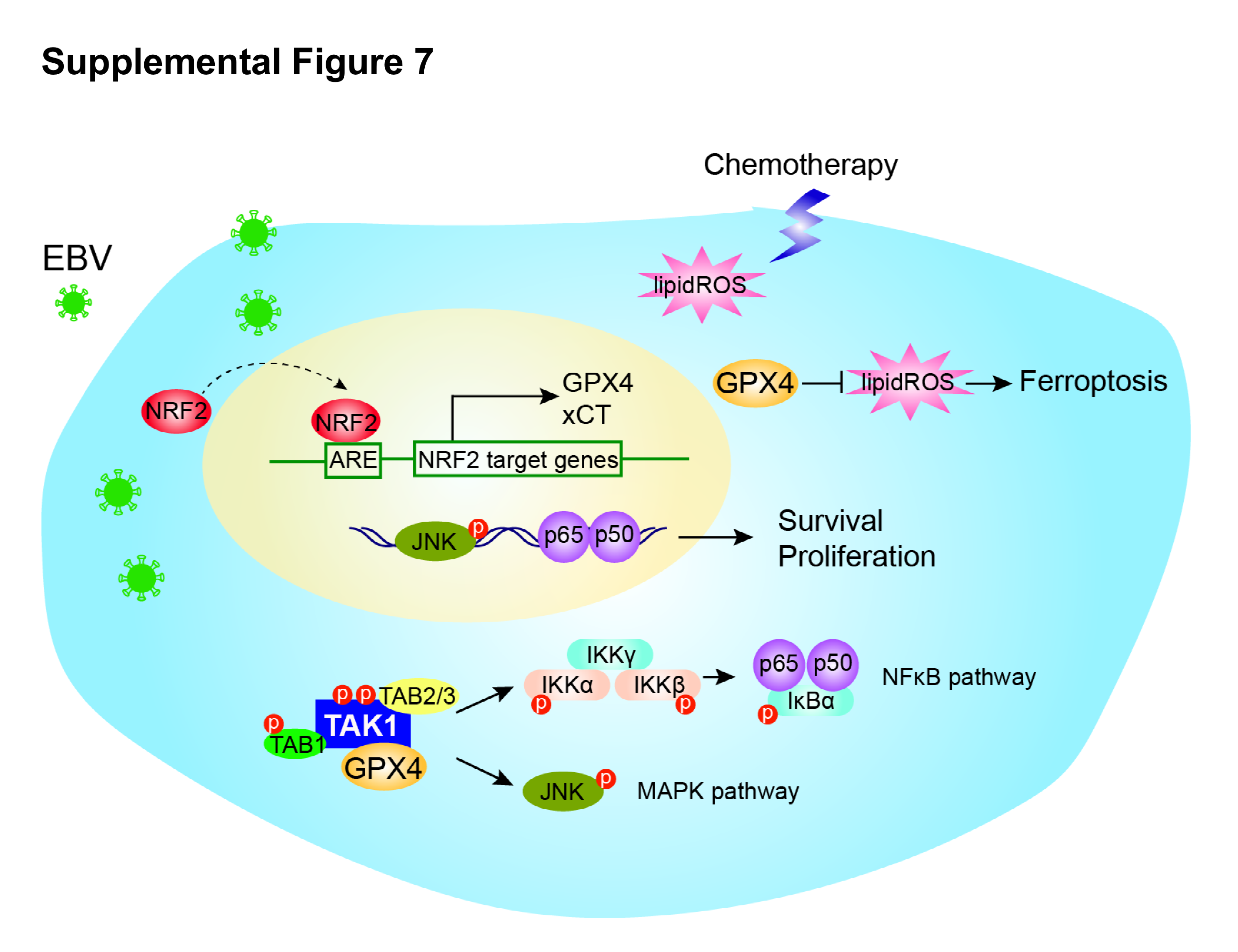

Supplement: Supplementary file 10 — Supplemental Figure 7 [file 41418_2022_939_MOESM10_ESM.png]
