# Supplementary material for: EBV infection-induced GPX4 promotes chemoresistance and tumor progression in nasopharyngeal carcinoma
Source: Cell Death Differ. 2022 Feb 1;29(8):1513–27. doi: 10.1038/s41418-022-00939-8 (PMC9346003; doi:10.1038/s41418-022-00939-8)
Supplement: Supplementary file 13 — Supplementary Table 3 [file 41418_2022_939_MOESM13_ESM.pdf]

Supplementary table 3. qRT-PCR primers

Gene	Primers
GPX4	Primer F: 5’- GAGGCAAGACCGAAGTAAACTAC-3’
	Primer R: 5’- CCGAACTGGTTACACGGGAA-3’
SCL7A11	Primer F: 5’- TCTCCAAAGGAGGTTACCTGC-3’
	Primer R: 5’- AGACTCCCCTCAGTAAAGTGAC-3’
EBNA1	Primer F: 5’-GTAGGGGATGCCGATTATTTTG-3’
	Primer R: 5’-CTCCTTGACCACGATGCTTTC-3’
LMP1	Primer F: 5’-GTATTGGCACAAGATGGAAAGC-3’
	Primer R: 5’-CAACTACCAGGCAGATGAGGC-3’
LMP2A	Primer F: 5’-ACGATGGCGGAAACAACTC-3’
	Primer R: 5’-GGGTCCTCATAAGGCGGTG-3’
BRLF1	Primer F: 5’-CGAGGACGGGATAGGTGAAC-3’
	Primer R: 5’-CGGCAAGCAGGTAGTGGAAC-3’
BZLF1	Primer F: 5’-CCCAGTCTCCGACATAACCC-3’
	Primer R: 5’-CAGGCTGTGGAACACCAATG-3’
NRF2	Primer F: 5'-TCAGCGACGGAAAGAGTATGA-3’
	Primer R: 5'-CCACTGGTTTCTGACTGGATGT-3’
